# Fluid proteomics of CSF and serum reveal important neuroinflammatory proteins in blood–brain barrier disruption and outcome prediction following severe traumatic brain injury: a prospective, observational study

**DOI:** 10.1186/s13054-021-03503-x

**Published:** 2021-03-12

**Authors:** Caroline Lindblad, Elisa Pin, David Just, Faiez Al Nimer, Peter Nilsson, Bo-Michael Bellander, Mikael Svensson, Fredrik Piehl, Eric Peter Thelin

**Affiliations:** 1 Department of Clinical Neuroscience, Karolinska Institutet, Stockholm, Sweden; 2 Division of Affinity Proteomics, Department of Protein Science, SciLifeLab, KTH-Royal Institute of Technology, Stockholm, Sweden; 3 Center for Neurology, Academic Specialist Center, Stockholm Health Services, Stockholm, Sweden; 4 Department of Neurosurgery, Karolinska University Hospital, Stockholm, Sweden; 5 Department of Neurology, Karolinska University Hospital, Stockholm, Sweden

**Keywords:** Traumatic brain injury, Protein biomarkers, Proteomics, Neuroinflammation, Blood–brain barrier, Apolipoprotein E4, Glasgow Outcome Score, Human

## Abstract

**Background:**

Severe traumatic brain injury (TBI) is associated with blood–brain barrier (BBB) disruption and a subsequent neuroinflammatory process. We aimed to perform a multiplex screening of brain enriched and inflammatory proteins in blood and cerebrospinal fluid (CSF) in order to study their role in BBB disruption, neuroinflammation and long-term functional outcome in TBI patients and healthy controls.

**Methods:**

We conducted a prospective, observational study on 90 severe TBI patients and 15 control subjects. Clinical outcome data, Glasgow Outcome Score, was collected after 6–12 months. We utilized a suspension bead antibody array analyzed on a FlexMap 3D Luminex platform to characterize 177 unique proteins in matched CSF and serum samples. In addition, we assessed BBB disruption using the CSF-serum albumin quotient (*Q*_A_), and performed Apolipoprotein E-genotyping as the latter has been linked to BBB function in the absence of trauma. We employed pathway-, cluster-, and proportional odds regression analyses. Key findings were validated in blood samples from an independent TBI cohort.

**Results:**

TBI patients had an upregulation of structural CNS and neuroinflammatory pathways in both CSF and serum. In total, 114 proteins correlated with *Q*_A_, among which the top-correlated proteins were complement proteins. A cluster analysis revealed protein levels to be strongly associated with BBB integrity, but not carriage of the Apolipoprotein E4-variant. Among cluster-derived proteins, innate immune pathways were upregulated. Forty unique proteins emanated as novel independent predictors of clinical outcome, that individually explained ~ 10% additional model variance. Among proteins significantly different between TBI patients with intact or disrupted BBB, complement C9 in CSF (*p* = 0.014, Δ*R*^2^ = 7.4%) and complement factor B in serum (*p* = 0.003, Δ*R*^2^ = 9.2%) were independent outcome predictors also following step-down modelling.

**Conclusions:**

This represents the largest concomitant CSF and serum proteomic profiling study so far reported in TBI, providing substantial support to the notion that neuroinflammatory markers, including complement activation, predicts BBB disruption and long-term outcome. Individual proteins identified here could potentially serve to refine current biomarker modelling or represent novel treatment targets in severe TBI.

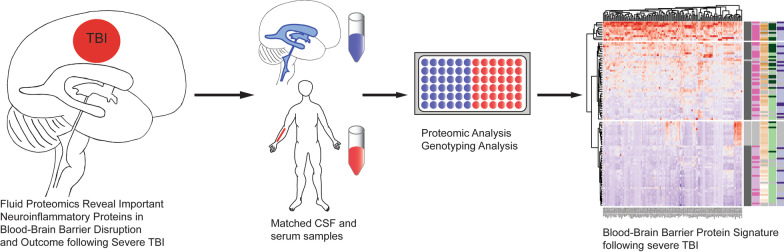

**Supplementary Information:**

The online version contains supplementary material available at 10.1186/s13054-021-03503-x.

## Introduction

Traumatic brain injury (TBI) is a common cause of death and acquired disability worldwide [1]. The initial trauma is followed by a series of secondary injury processes, which may lead to deterioration and irreversible brain damage [2]. Increased knowledge of these might be of key relevance for long-term outcome and improved patient management. Among secondary injury pathologies, blood–brain barrier (BBB) disruption is of particular interest. The acute, mechanically-induced BBB injury has been shown to peak at 1–3 h post-TBI [3, 4] and contribute to the inflammatory activation of (CNS) inherent cells, such as astrocytes and microglia, but also in facilitating the infiltration of immune cells from the systemic circulation [5, 6]. This generates an inflammatory cascade that can exacerbate BBB injury, thereby increasing the intensity of CNS neuroinflammation [7]. Jointly, BBB injury and neuroinflammation propagate secondary injury pathologies, such as edema development, increased intracranial pressure, decreased cerebral perfusion, and consequent ischemia [4], presumably of importance for long-term outcome. It is unclear whether these acute TBI processes are influenced by the genetic set up, but in the absence of trauma the E4 variant of apolipoprotein E (APOE4) is associated with reduced BBB function and predicts risks of cognitive decline [8].

Even though there are radiological techniques that quantitatively assess BBB disruption [9], the current gold-standard metric within the field of clinical neuroscience is the cerebrospinal fluid (CSF) to blood albumin quotient (*Q*_A_) [10]. An increased *Q*_A_ indicates albumin leakage due to loss of BBB integrity. Following TBI, *Q*_A_ has shown to be associated with both structural [11], and neuroinflammatory [12–14] proteins, important as albumin does not confer information on underlying pathophysiology. Yet, as these studies included only a small selection of proteins, they potentially miss out on important biological information, pertaining to protein families and pathways that might confer joint or discrepant functions within the CNS. More comprehensive proteomic profiling efforts are warranted to deduce the pathophysiology causing BBB disruption [15, 16].

Mass-spectrometry holds the largest capacity for simultaneous assessment of multiple proteins [17] and has been utilized in numerous TBI studies [18–26]. Inherent limitations of mass-spectrometry entail its limited capacity to detect low-abundance proteins (e.g. cytokines) [17], thus obstructing detection of low- and high-abundant proteins within the same study. An alternative technique is affinity proteomics, combining microarray technology with affinity reagents [27] that is suitable for multiplexed protein screens in large numbers of samples [28, 29] from both serum [28, 29] and CSF [27]. These broad advantages of affinity proteomics have not yet been utilized in the clinical TBI setting.

Collectively, although BBB disruption seems to be a key secondary injury event ensuing TBI, no systematic assessment of *Q*_A_ related protein alterations has yet been described. We therefore conducted a proteomic screen of neuroinflammatory, BBB-related, and CNS structural proteins in CSF and serum of neuro-critical care unit (NCCU) treated TBI patients and controls utilizing affinity-based proteomics, while also analyzing APOE4. The main objective was to determine to what degree changes in protein concentrations could be associated to BBB disruption, as well as their association with long-term outcome following severe TBI.

## Methods

This was a prospective, observational study, part of two separate studies conducted at the Karolinska University Hospital, and Karolinska Institutet, Stockholm, Sweden. The first study included TBI patients between 2007 and 2015. Oral informed consent was granted by next-of-kin. The second study included healthy volunteers, used as control subjects here, between 2014 and 2015. All control subjects provided written, informed consent. All research activities were in accordance with Swedish law and the Declaration of Helsinki. Ethical approvals (#2005/1526-31/2; #2014/1201-31/1) were granted by the Swedish Ethical Review Authority.

### Study participant inclusion and exclusion criteria

Inclusion criteria for TBI patients were: (1) severe TBI (as per Glasgow Coma Scale (GCS) 3–8 upon hospital admission or else a higher GCS score but with a significant risk for deterioration) in need of NCCU treatment and invasive intracranial monitoring, and (2) age 18–75 years. Exclusion criteria comprised: (1) desolate prognosis precluding NCCU treatment, (2) penetrating TBI, (3) unconsciousness due to etiology other than TBI, (4) underlying chronical condition precluding follow-up, or (5) other reason precluding follow-up. Inclusion criteria for control subjects were: (1) previously healthy, (2) age 18–50 years, (3) sufficient linguistic knowledge to participate in self-evaluation forms. Exclusion criteria were: (1) ongoing, or history of, psychiatric illness, (2) family history of serious psychiatric comorbidity, (3) somatic illness precluding physical activity, (4) current pharmacological treatment interacting with the study intervention, (5) substance abuse (smoking or narcotic substances), or (6) pregnancy. Sample size calculation was based on expected protein level difference between TBI patients and control subjects and was exerted as a two-sample t-test. We utilized Cohen’s d [30, 31] as effect size metric and set it to 0.8 (large effect) [30, 31] in a power calculation utilizing the R package pwr [32]. In order to obtain 80% power at the 0.05 significance level with* n* = 15 control patients, we needed to recruit* n* = 77 TBI patients. As this was not based on empirical data, we included patients continuously throughout the study period.

### Clinical management, data, and sample acquisition

NCCU management of severe TBI at Karolinska University Hospital has been described elsewhere [33]. In brief, Karolinska University Hospital employs an intracranial pressure (ICP-) driven approach, in accordance with the Brain Trauma Foundation Guidelines [34]. ICP is monitored either through a closed external ventricular drain (EVD) (Medtronic, USA), or an intraparenchymal pressure monitor (Codman & Shurtleff Inc. Raynham, MA, USA or Rehau AG + CO, Rehay, Germany). While EVDs may be used to drain CSF in order to decrease ICP, the choice between monitoring device is multifactorial and not exclusively reliant on injury severity. At the NCCU, multi-modal monitoring data is automatically collected. Through the Karolinska University Hospital TBI Database, additional data is collected prospectively and comprise neurological variables, injury severity score variables, radiological variables, and outcome data, described in detail elsewhere [11]. Functional outcome data (Glasgow Outcome Score, GOS) was collected at 6–12 months following hospital discharge, through structured questionnaires, or follow-up assessments in the outpatient clinic at the Neurosurgical Department. We collected CSF and serum, used for APOE genotyping, proteomic, and albumin analysis. The latter was assessed as *Q*_A_, i.e. the CSF/serum albumin quotient [10], with the reference intervals [35]: 15–29 years < 0.006; 30–49 years < 0.007; and ≥ 50 years < 0.009. Sampling time points were not identical for albumin_CSF_, albumin_serum_ and the proteomic samples from CSF and serum. Time discrepancies were in median (interquartile range [IQR]): 4.3 (0–11.8) hours for albumin_CSF_ and albumin_serum_ samples; 0.88 (− 2.27 to 9.15) hours for albumin_CSF_ and the proteomic sample; and − 2.83 (− 3.82 to − 2.08) hours for albumin_serum_ and the proteomic sample.

#### Sample acquisition

Control subjects were recruited to a study on effects of a physical exercise intervention [36], of which only baseline samples were used. Participants were instructed to abstain from physical exercise 7 days before sampling, performed by lumbar puncture and venipuncture, between 7.30 and 9 AM while fasting since midnight after a full night of bed rest. For TBI patients, blood was sampled through an arterial line and CSF through an EVD. TBI sample acquisition occurred in median at 60.8 h (IQR 36.6–109.1) following trauma for CSF samples and 53.3 h (30.5–91.1) for serum samples (Additional file 1: Figure S1A). Samples were stored locally in 4 °C in median 1 day (0–1) for both CSF and serum (Additional file 1: Figure S1B), until delivery to a local biobank, where samples were vertically incubated for 30 min before centrifugation for 15 min at 2000 g, aliquoting, and storage at − 80 °C until further analysis [37]. No protein content alteration was seen per sample (Additional file 2: Figure S2A) or analyte (Additional file 2: Figure S2B, representative example) due to delayed biobank delivery.

### Genotyping

Whole blood was collected together with serum in ethylenediaminetetraacetic acid (EDTA) tubes, and was frozen in the biobank until DNA extraction. Genotyping was performed with the SNP markers rs429358 (ApoE112) and rs7412 (ApoE158) using single base primer extension (SBE) with detection of the incorporated allele by ¨Fluorescent Polarization Template Dye Incorporation¨ (FP-TDI) [38]. Signal intensities were read using a Tecan Genios Pro fluorescence absorbance reader. Raw data from the fluorescence polarization was converted to genotype data using the software AlleleCaller 4.0.0.1 and alleles ε2, ε3 or ε4 were identified.

### Proteomic analysis

In total, 177 protein depicted through 220 antibodies were examined (Additional file 3: Table S1, where the full protein name is provided). For 43 proteins, two antibodies targeted different regions of the same protein, i.e. sibling antibodies [39]. The protein panel was chosen based on CNS-enrichment [40], previous clinical/experimental/mass-spectrometry TBI studies, or previous neuroinflammation studies [20, 24, 26, 41–45]. Antibodies were selected from the Human Protein Atlas (HPA) (www.proteinatlas.org) [46].

Antibodies were immobilized onto color-coded magnetic beads (MagPlex, Luminex Corporation) as previously described [28]. Briefly, the beads surface was activated by using 0.1 M sodium hydrogen phosphate (Sigma), 0.5 mg of N-hydroxysulfosuccinimide (sulfo-NHS) (Nordic Biolabs) and 0.5 mg 1-ethyl-3-(3-dimethylaminopropyl)carbodiimide hydrochloride (EDC) (ProteoChem). Beads were then incubated with antibodies (16 μg/ml in 2-(*N*-morpholino)ethanesulfonic acid [MES] buffer, Sigma) for 2 h at room temperature. Each antibody type was immobilized on a different bead identity (bead type with specific color-code). After incubation, the beads were washed with phosphate-buffered saline (PBS, Fisher Scientific) 0.05% Tween-20 (Fisher Scientific) (PBS-T) to eliminate the antibody excess, stored overnight in blocking buffer (Roche blocking reagent for ELISA, Roche), and combined into a suspension bead array.

Samples were processed as previously described, with minor adjustments [27, 47]. Serum and CSF samples were separately randomized into 96-well microtiter plates. CSF samples were diluted 0.6:1 in PBS (Fisher Scientific) with 0.5% bovine serum albumin (BSA, Sigma), 0.1% rabbit IgG (Nordic Biosite), and labeled with biotin (Fisher Scientific). The samples were then further diluted 1:8 in assay buffer (0.1% casein (Fisher Scientific), 0.5% polyvinyl alcohol (Sigma), 0.8% polyvinylpyrrolidone (Sigma) in PBS-T (0.05% Tween-20 (Fisher Scientific)), supplemented with 0.5 mg/ml rabbit IgG (Nordic Biosite)), heat treated (56 °C for 30 min), and incubated with the bead array overnight at room temperature. Serum samples were diluted 1:10 in PBS (Fisher Scientific) prior to labeling with biotin (Fisher Scientific), and further diluted 1:50 in assay buffer (0.1% casein (Fisher Scientific), 0.5% polyvinyl alcohol (Sigma), 0.8% polyvinylpyrrolidone (Sigma) in PBS-T (0.05% Tween-20 (Fisher Scientific)), supplemented with 0.5 mg/ml rabbit IgG (Nordic Biosite)) after labeling, heat treated (56 °C for 30 min), and incubated with the bead array for 2 h at room temperature.

The captured proteins were cross-linked to the antibodies for 10 min at room temperature using 0.4% paraformaldehyde (Thermo Scientific). The antibody-protein immunocomplexes were detected by using a streptavidin-conjugated phycoerythrine (Fisher Scientific) and a FlexMap3D instrument (Luminex Corporation). The relative protein abundance was reported as median fluorescence intensity (MFI) for each bead identity and sample. Quality control assessments are described in Additional file 4. Briefly, bead counts were evaluated per sample and analyte (Additional file 5: Figure S3A-S3B). Due to a small systematic increase in MFI_CSF_ samples (Additional file 6: Figure S4A), background subtraction was conducted (Additional file 6: Figure S4B). MFI values varied across analytes (Additional file 6: Figure S4C), of which one was excluded due to borderline non-detected signal (Additional file 6: Figure S4C, inset). Antigen profiles were assessed per sample and analyte (Additional file 7: Figure S5, Additional file 8: Figure S6, Additional file 3: Table S2), resulting in the exclusion of a few sibling antibodies (Additional file 4).

### Statistical analysis

For inferential analysis, matched CSF-serum patient samples were compared. Validation analysis was exerted in the non-matched TBI cohort with serum-samples only. We used R (version 4.0.2) [48], through RStudio® (version 1.3.1056) and the tidyverse [49], RColorBrewer [50], cowplot [51], and gridExtra [52] packages. Continuous data were presented as median (IQR). Categorical data were presented as count (%). For multiple testing correction, we used the Bonferroni, Holm [53] or the false-discovery rate (FDR) [54] method. A *p* value < 0.05 was considered significant, unless otherwise stated.

A few variables (pre-hospital hypotension, *Q*_A_, APOE allele status) had a substantial number of missing values (Table 1, Additional file 9: Figure S7). When applicable, we conducted multiple imputation using* n* = 200 imputations in the mice package [55]. Reported *p* values were calculated as the unadjusted median *p* value from all imputations.

**Table 1:** Study participant demography

Variable	TBI cohort (matched samples) *n* = 90	Control cohort *n* = 15	TBI validation cohort (non-matched samples) *n* = 96	Unit/metric
Age	57 (41–62)	25 (22–29)	54 (36.5–63)	Years
Male	67 (74)	7 (47)	72 (75)	count (%)
GCS admission	7 (3–9)		6 (3–11)	Scale 1–15
GCS motor admission	4 (1–5)		4 (1–5)	Scale 1–6
Pupils	Bilaterally responsive: 67 (74)		Bilaterally responsive: 69 (72)	Count (%)
	Unilaterally unresponsive: 11 (12)		Unilaterally unresponsive: 9 (9.4)	
	Bilaterally unresponsive: 9 (10)		Bilaterally unresponsive: 16 (17)	
	Missing: 3 (3.3)		Missing: 2 (2.1)	
Head AIS	1 (minor): 0 (0)		1 (minor): 0 (0)	Score 1–6
	2 (moderate): 0 (0)		2 (moderate): 1 (1.0)	
	3 (serious): 10 (11)		3 (serious): 10 (10.4)	
	4 (severe): 30 (33)		4 (severe): 25 (26)	
	5 (critical): 43 (48)		5 (critical): 57 (59)	
	6 (maximum): 0 (0)		6 (maximum): 0 (0)	
	Missing: 7 (7.8)		Missing: 3 (3.1)	
ISS	25 (19–29)		25 (17–29)	Scale
	Missing: 7 (7.78)		Missing: 3 (3.1)	
Multitrauma	29 (32)		26 (27)	Count (%)
Hypotension at SoA	2 (2.2)		1 (1.0)	Count (%)
	Missing: 24 (27)		Missing: 26 (27)	
Hypoxia at SoA	15 (17)		18 (19)	Count (%)
	Missing: 4 (4.4)		Missing: 4 (4.2)	
Stockholm CT score	2.5 (2–3.3)		2.5 (1.9–3.3)	Scale
*Q*_A_	0.0041 (0.0018–0.011)	0.0040 (0.0035–0.0060)	0.009 (0.003–0.027)	Quotient
	Missing: 19 (21.1)		Missing: 71 (74)	
APOE allele status	No allele: 57 (63)		No allele: 64 (67)	Count (%)
	Heterozygote: 16 (18)		Heterozygote: 17 (18)	
	Homozygote: 2 (2)		Homozygote: 3 (3.1)	
	Missing: 15 (17)		Missing: 12 (12.5)	
Evacuation aSDH	33 (38)		50 (61)	
Evacuation EDH	6 (6.8)		14 (17)	
Evacuation tICH	24 (27)		13 (16)	
ICP monitor^a^	38 (43)		60 (73)	
Other intracranial monitoration^b^	55 (63)		39 (48)	
GOS	GOS 1 (death): 12 (13)		GOS 1 (death): 10 (10.4)	Score 1–5
	GOS 2 (vegetative): 0 (0)		GOS 2 (vegetative): 1 (1.0)	
	GOS 3 (severe disability): 34 (38)		GOS 3 (severe disability): 30 (31)	
	GOS 4 (moderate disability): 28 (31)		GOS 4 (moderate disability): 29 (30)	
	GOS 5 (good recovery): 16 (18)		GOS 5 (good recovery): 26 (27)	
Unfavorable GOS	GOS 1–3: 46 (51)		GOS 1–2: 41 (43)	count (%)

Patient demographics are summarized for all cohorts. Data is depicted as median (interquartile range [IQR]) if continuous and otherwise as count (%)

*AIS* Abbreviated Injury Scale, *APOE* ApoE lipoprotein, *aSDH* acute subdural hematoma, *CT* computerized tomography, *EDH* epidural hematoma, *EVD* external ventricular drain, *GCS* Glasgow Coma Scale, *ICP* intracranial pressure, *ISS* injury severity score, *GOS* Glasgow Outcome Scale, *Q*_*A*_ albumin quotient, *SoA* site of accident, *tICH* traumatic intracerebral hemorrhage

^a^Other than EVD

^b^Other than EVD or ICP monitor

#### Protein characterization

Analytes were characterized using the HPA [46, 56] version 19.1 (release date 2019/12/19, Ensembl version 92.38), using the protein tissue data, RNA tissue data (Consensus data set), and Brain Atlas [57] RNA data (Additional file 4).

#### Parallel assessments in CSF, serum, and relationship with BBB disruption

T-distributed stochastic neighbor embedding (t-SNE) [58, 59] was employed to examine if proteins pertained to compartment (CSF or blood) and disease characteristics among study subjects (Additional file 4). We assessed protein levels in CSF and serum under control conditions and following TBI using the Wilcoxon rank sum test (FDR, *p*_adjusted_ < 0.05) and the Wilcoxon signed rank test (FDR, *p*_adjusted_ < 0.01).

Cluster analysis within CSF and serum was conducted for proteins that had a CSF/serum ratio significantly correlated (Kendall correlation, Holm method, *p*_adjusted_ < 0.05) with *Q*_A_ (Additional file 4). Clusters were visualized using the ComplexHeatmap package [60]. Proteins significant upon linear regression (FDR, *p*_adjusted_ ≤ 0.01) compared with the reference cluster (containing the majority of control patients) were deemed significantly altered. For CSF (*n* = 3 clusters), proteins needed to be concurrently significant in all clusters compared with the reference cluster. Protein levels between TBI patients with disrupted/intact BBB were compared using the Wilcoxon Rank Sum Test (FDR, *p*_adjusted_ < 0.05). Linear regression models were used to examine if APOE4 carriership was important for *Q*_A_, or protein levels (FDR, *p* ≤ 0.05). Age, gender and injury scores were used as covariates in addition to APOE4 variant.

#### Pathway and outcome analysis

Pathway analysis through the pathfindR package [61] and pipeline [62], was conducted for proteins altered following TBI or that pertained to a BBB integrity related cluster. For protein input, *p* value thresholds were set to 0.05. For enrichment analyses, the Biocarta gene set and the Bonferroni method (*p*_adjusted_ ≤ 0.05) for multiple correction were used.

Proteins of interest for outcome analysis were: (1) protein intersects between CSF cluster analysis and TBI-induced altered proteins in CSF, (2) protein intersects between CSF cluster analysis and TBI-induced altered proteins in serum, and (3) significantly elevated/decreased proteins following BBB disruption. Protein intersects were visualized using the VennDiagram package [63] in R. We used GOS as dependent variable and protein levels of an individual protein (or other variable of interest such as *Q*_A_) as independent variable in a proportional odds regression analysis, using the rms package [64]. Only TBI patients were included, as healthy control subjects by definition had no GOS data. We conducted univariable analysis, and if significant (FDR, *p*_adjusted_ ≤ 0.05 or ≤ 0.01 if multiple testing, the latter for dichotomized GOS/short-term mortality), multivariable analysis (FDR, *p*_adjusted_ < 0.05 if multiple testing or p_imputed_ ≤ 0.05 if imputed). We used age, GCS motor score, pupillary reactions, hypoxia, hypotension and Stockholm computerized tomography (CT) score as covariates in accordance with the International Mission for Prognosis and Clinical Trial (IMPACT) database studies [65]. We used the Stockholm instead of the Marshall CT score, as the former has been shown to be superior [66, 67]. When applicable, we conducted step-down modelling to see how the proteins performed jointly in the regression models.

## Results

### Patient demographics

In total, 190 NCCU TBI patients and 15 control patients were included. Of these, *n* = 4 TBI patients were excluded due to low bead counts (Additional file 5: Figure S3A). Of the remaining, data analysis was conducted on the 90 TBI patients and 15 healthy controls that had matched CSF and serum samples. The *n* = 96 TBI patients that merely had serum samples were used for validation analyses and are referred to as the “validation cohort”. Patient demography is depicted in Table 1. TBI patients comprised predominantly middle-aged men among whom *n* = 2 (2%) were homozygotes for APOE4. Even though 32% of patients suffered a multi-trauma, the CNS trauma was the dominant pathology as deemed by a head-Abbreviated Injury Score (AIS) of 5 (“critical”) among 48% of patients. In total, 51% of patients suffered an unfavorable outcome (GOS 1–3). TBI patients and the validation cohort differed in type of surgery performed and long-term prognosis. Notably, while all patients in the TBI cohort had EVDs, the validation cohort had fewer (*n* = 25, 31%), but higher degree of intraparenchymal ICP monitors.

### Protein characterization

**Fig. 1:**
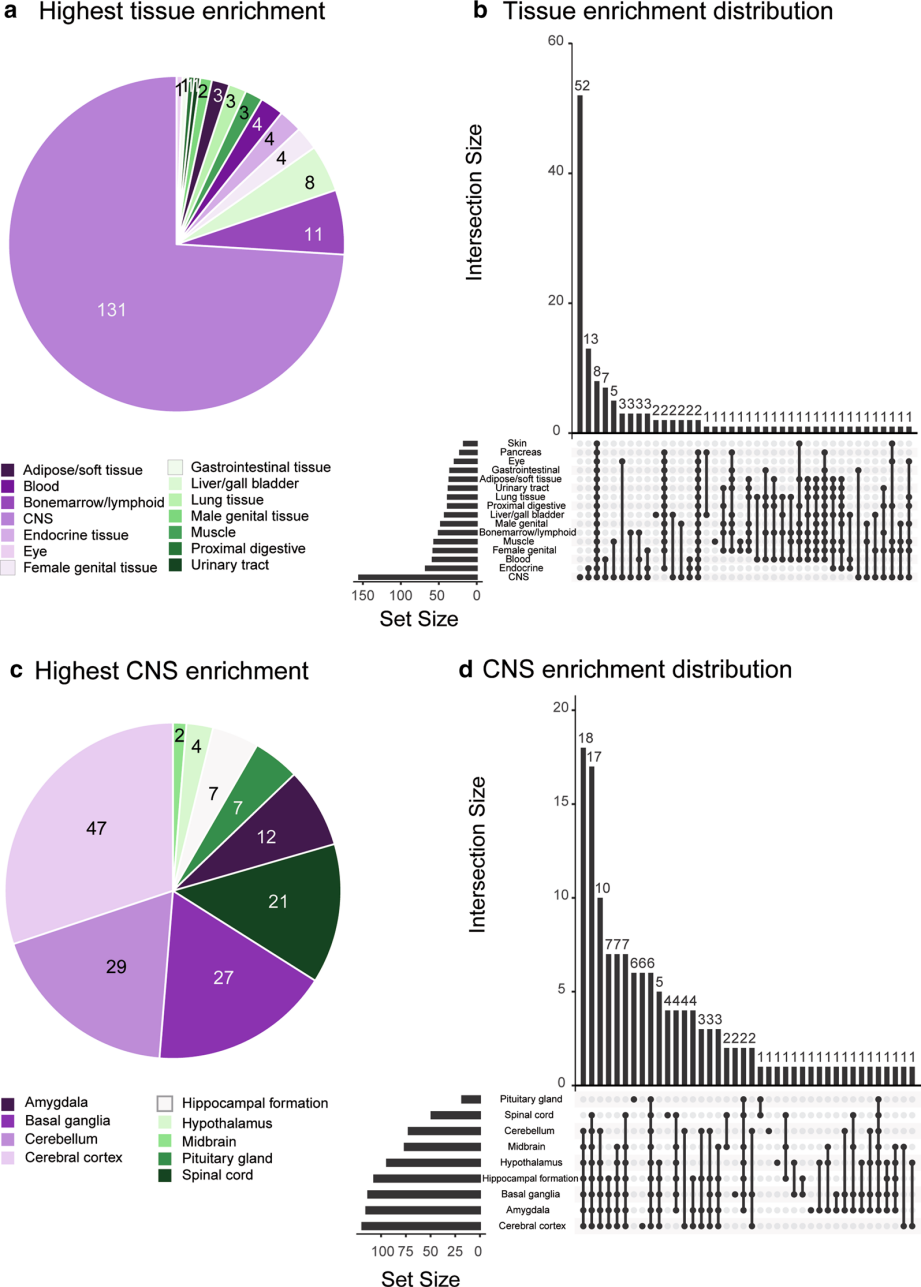
Assessed proteins were predominantly CNS structural proteins. The vast majority of proteins exhibited highest tissue enrichment in the CNS, with the second most frequent category being immune-system organs (**a**). Notably, numerous proteins were concomitantly expressed in multiple tissues (**b**). Within the Brain Atlas, the majority were cerebral cortex enriched (**c**), but few proteins were exclusively expressed within one CNS-niche (**d**). Protein characterization data was obtained from the Human Protein Atlas. *CNS* central nervous system

The majority of proteins exhibited highest tissue enrichment in the CNS (Fig. 1a), although several proteins exhibited high RNA expression in multiple different tissues (Fig. 1b). Within the Brain Atlas, proteins exhibited top RNA expression in the cerebral cortex proteins (Fig. 1c), but concurrent CNS tissue expression was common (Fig. 1d).

### TBI alters CSF and serum protein levels and upregulates neuroinflammatory pathways

**Fig. 2:**
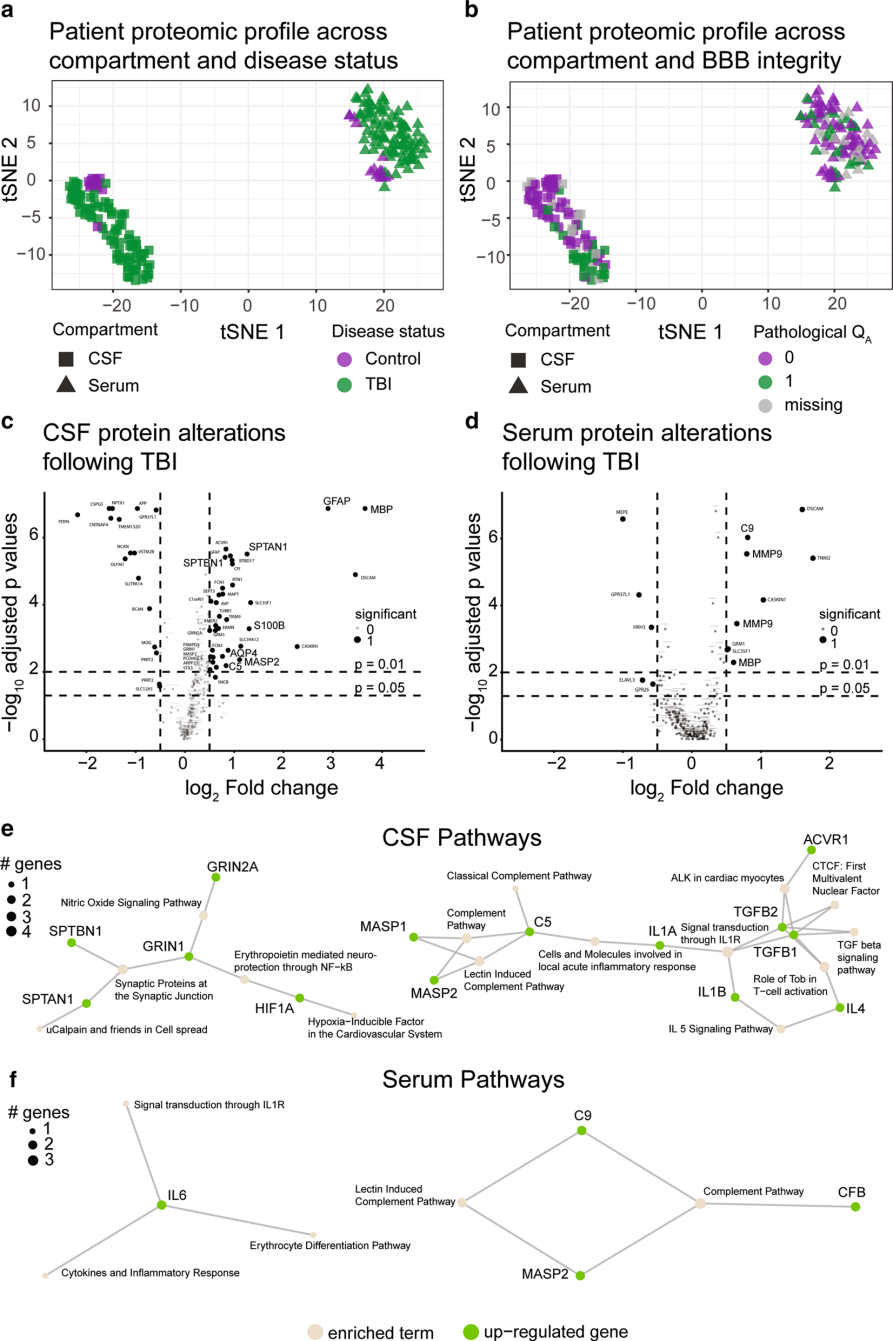
A severe TBI induces protein alterations in CSF and serum. Individual patient proteomic profiles were different in CSF compared with serum, utilizing tSNE. Following a severe TBI, additional proteomic alterations occur within both of these compartments (**a**). Individual patient attributes, such as BBB disruption, seemed associated with some of TBI patient heterogeneity, predominantly in CSF (**b**). At the individual protein level, this was mimicked by altered protein levels in both CSF and serum (**c**, **d**). Graphical significance threshold was set to log_2_ FC |0.5| and adjusted *p* value < 0.05, and values not fulfilling these criteria were diminished in size and shaded in light-gray. In CSF, both CNS structural and neuroinflammatory protein levels were increased following a severe TBI (**c**). This was reflected in pathway upregulations of structural, metabolic, and inflammatory pathways (**e**). In contrast, fewer protein were altered in serum (**d**), and upregulated pathways were predominantly neuroinflammatory (**f**). *CSF* cerebrospinal fluid, *TBI* traumatic brain injury, *tSNE* t-distributed stochastic neighbor embedding. All full protein names are given in Additional file 3: Table S1

Among control subjects, CNS-originating proteins (e.g. GAP43, log_2_ fold change [FC] 3.41, *p* < 0.001) were enriched in CSF, while for example complement proteins (e.g. C1QB, log_2_ FC − 2.38, *p* < 0.001) were enriched in serum (Additional file 10: Figure S8). Following TBI, t-SNE demonstrated that the patients’ protein composition grouped along compartment (serum and CSF) and disease status (TBI and control) (Fig. 2a). t-SNE 2 seemed related to BBB integrity in CSF (Fig. 2b). This indicates that the CSF and serum proteomes are distinct in health and following TBI, and that injury characteristics may be reflected in protein composition. In fact, following TBI, *n* = 124 (unique) proteins were altered in either CSF or serum compared with controls (Fig. 2c, d, Additional file 3: Table S3). This allowed assessment of currently used TBI biomarkers, comprising the astrocytic proteins S100B and glial fibrillary acidic protein (GFAP), as well as the neuronal proteins neuron-specific enolase (NSE, or ENO2), neurofilament-light (NFL), and ubiquitin carboxy-terminal hydrolase-L1 (UCH-L1) [37]. We could confirm previous findings of upregulation of S100B, GFAP, NSE (ENO2), and NFL post-TBI (Additional file 3: Table S3).

Following TBI, far more proteins were altered in CSF (*n* = 109) than in serum (*n* = 35). In CSF, *n* = 81 (74%) of all altered proteins were CNS related, whereas *n* = 11 (10%) were immune system related. Proteins enriched in CSF following TBI were among else myelin basic protein (MBP) (ΔMFI = 3655, *p* < 0.001), and AQP4 (ΔMFI = 2208, *p* = 0.002). Similarly to CSF, the majority of altered proteins in serum were CNS related (*n* = 23, 66%), whereas *n* = 7 (20%) proteins were immune system related. The proteins in serum that exhibited the highest ΔMFI were the complement proteins CFB (ΔMFI = 2131, *p* < 0.001) and C9 (ΔMFI = 2000, *p* < 0.001). Top-altered pathways in CSF included the lectin-induced complement pathway, erythropoietin-mediated neuroprotection through Nuclear Factor Kappa-Light-Chain-Enhancer of Activated B cells (NF-κB), synaptic proteins at the synaptic junction, and Role of Tob in T-cell activation (Fig. 2e). This was partially mimicked in serum with regard to the neuroinflammatory pathways, particularly the complement system (Fig. 2f), which also held true for our validation cohort (Additional file 11: Figure S9). Surprisingly, merely *n* = 19 proteins were concurrently altered in both CSF and serum following TBI. Among these, *n* = 12 proteins (63%) were CNS enriched and *n* = 4 (21%) immune system related. Among immune system proteins, notably all but one (CXCL1) were complement system proteins (CFI, FCN1, MASP2).

### BBB disruption yields a protein signature in CSF and is predictive of outcome

**Fig. 3:**
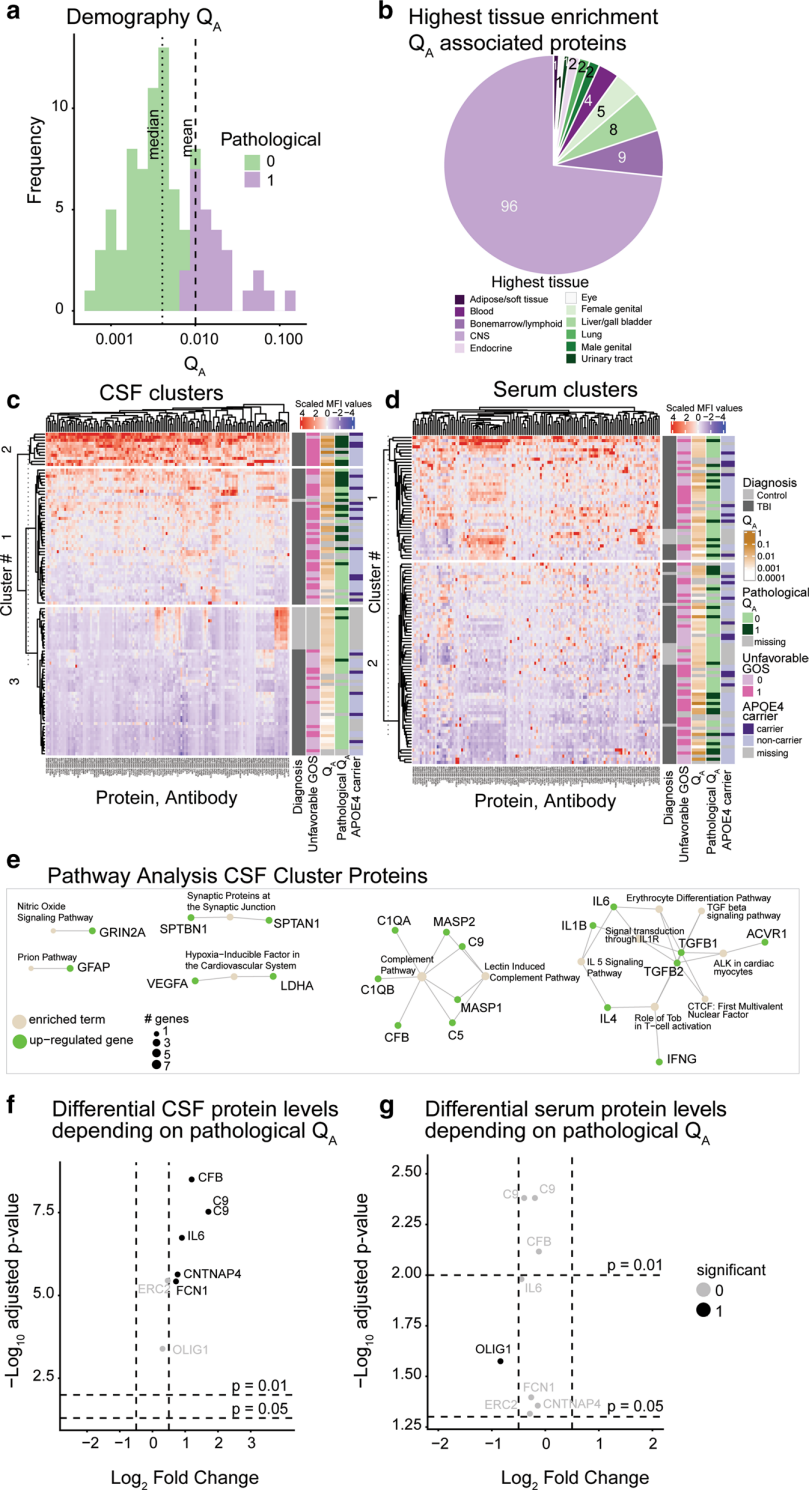
BBB disruption co-occurs with upregulation of innate immune pathways, notably the complement cascade. A severe TBI elicited an acute BBB disruption among a subset of patients, quantified using *Q*_A_ (**a**). Among the *n* = 114 proteins significantly correlated with *Q*_A_, the majority were nervous system or immune system enriched (**b**). Using hierarchical clustering on CSF and serum protein measurements respectively, protein levels clearly clustered depending on BBB integrity status in CSF (**c**), but less so in serum (**d**). APOE carrier status was not associated with protein levels in either group (**c**, **d**). In CSF, this corresponded to pathway upregulation of predominantly innate immune mechanisms (**e**). Examining proteomic profiles between patients with disrupted and intact BBB, a handful of proteins were significant in CSF (**f**) and merely one in serum (**g**). *APOE* Apolipoprotein E, *CSF* cerebrospinal fluid, *CNS* central nervous system, *GOS* Glasgow Outcome Score, *MFI* median fluorescence intensity, *Q*_*A*_ albumin quotient, *TBI* traumatic brain injury. All full protein names are given in Additional file 3: Table S1

Under homeostasis, the amount of ventricular albumin comprises ~ 40% of lumbar albumin [68] and the *Q*_A_ reference interval is defined for lumbar albumin [35]. In line with our previous work we did not attempt any rostro-caudal correction for the *Q*_A_ values [11, 14], as ventricular albumin is expected to be higher than the lumbar ditto following a supratentorial trauma. As expected [69], a few control subjects exhibited pathological *Q*_A_ values (Table 1). In contrast, BBB disruption was present among *n* = 23 TBI patients (32%), and median *Q*_A_ was 0.004 (0.002–0.011) (Fig. 3a). *Q*_A_ was an independent significant predictor of GOS (*p* = 0.044, ΔNagelkerke’s pseudo-R^2^ = 8.89%). This finding is novel and highlight BBB disruption as a prognostic marker for severe TBI. This finding could not be attributed to multi-trauma as multi-trauma patients had slightly lower *Q*_A_ values (*p* = 0.035), and *Q*_A_ was negatively correlated with multi-trauma (ρ_Spearman_ = − 0.25). APOE4 variant was not associated with *Q*_A_ adjusted for age and sex (*p* = 0.494), or if injury severity was added to the model (*p* = 0.634).

In total, 114 unique CSF/serum protein ratios correlated significantly with *Q*_A_, conferring a median correlation coefficient τ 0.33 (0.29–0.40) (Additional file 3: Table S4). The ten proteins with highest correlation coefficient τ between CSF/serum ratio and *Q*_A_ were complement proteins, except VCAM1 (Table 2). The majority of proteins that correlated with *Q*_A_ were either nervous system or immune system proteins (Fig. 3b, Additional file 3: Table S4). Protein size had no obvious relationship with protein levels associated with *Q*_A_ (Table 2). APOE4 was not a predictor of the *Q*_A_ associated protein levels in either CSF or serum.

**Table 2:** Complement proteins exhibited highest correlations with *Q*_A_

Protein, antibody	Specific function	Protein size [kDa]	τ	adjusted *p* value
C1QB HPA052116	Innate immunity/complement system	26.7	0.67	< 0.001
CFB HPA001817	Innate immunity/complement system	85.5	0.66	< 0.001
C9 HPA029577	Innate immunity/complement system	63.2	0.65	< 0.001
C9 HPA070709	Innate immunity/complement system	63.2	0.65	< 0.001
C1QA HPA002350	Innate immunity/complement system	26	0.64	< 0.001
MASP2 HPA029314	Innate immunity/complement system	75.7	0.58	< 0.001
VCAM1 HPA069867	Cell cell communication	81.3	0.54	< 0.001
FCN3 HPA071173	Innate immunity/complement system	31.7–32.9^a^	0.54	< 0.001
MASP2 HPA029313	Innate immunity/complement system	75.7	0.52	< 0.001
C5 HPA075945	Innate immunity/complement system	188.3	0.52	< 0.001

Top 10 *Q*_A_ correlated proteins as deemed by correlation coefficient Kendall τ. Correlations were calculated between protein CSF/serum ratio and *Q*_A_. Protein sizes were derived from the Human Protein Atlas for the specific protein splice variant represented by the Human Protein Atlas antibody

*CNS* central nervous system, *CSF* cerebrospinal fluid, *Da* Dalton, *Q*_*A*_ albumin quotient. Full protein names are detailed in Additional file 3: Table S1

^a^Protein size interval provided as Human Protein Atlas antibody is not specified for protein splice variant

Cluster analysis of *Q*_A_ correlated proteins demonstrated that protein levels paralleled *Q*_A_ in CSF, but *not* in serum (Fig. 3c, d). The protein levels exhibited an association with dichotomized GOS (in CSF), but not APOE4 (Fig. 3c, d). Among proteins significantly different between CSF clusters, pathway analysis exhibited that structural and inflammatory pathways were upregulated (Fig. 3e). Merely *n* = 7 of all *Q*_A_ associated proteins were altered dependent on intact or disrupted BBB. In CSF, the majority of these proteins were inflammatory (CFB, C9, IL6, FCN1), whereas the sole significant protein in serum was the structural protein OLIG1 (Fig. 3f, g).

### Proteins associated with BBB disruption are outcome predictors following TBI

**Fig. 4:**
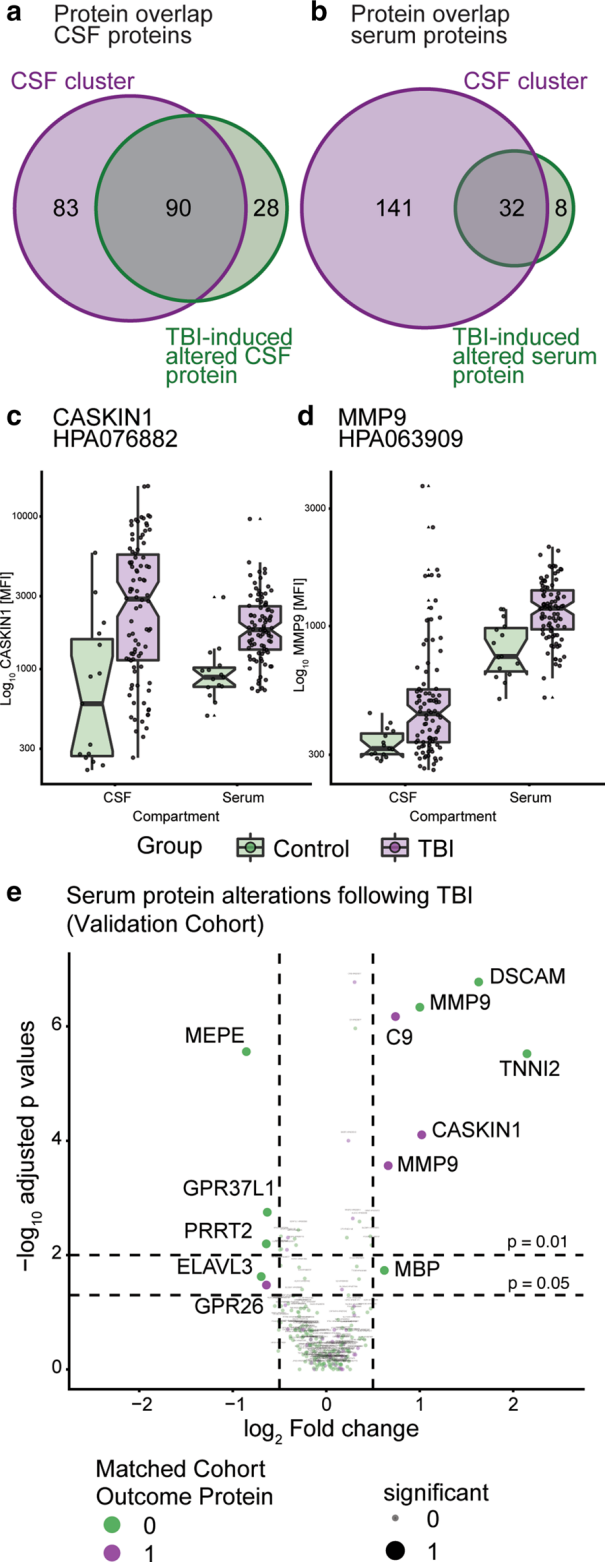
Proteins associated with BBB disruption and TBI-induced protein level alterations were outcome predictors following TBI. Using the hierarchical clustering depicted in Fig. 3d, *Q*_A_ associated proteins significantly different between clusters were derived. Of these, *n* = 90 proteins were found to overlap with proteins altered in CSF following TBI as portrayed in Fig. 2c (**a**). Similar assessments between CSF clusters and TBI-induced protein alterations in serum yielded an overlap of *n* = 32 proteins (**b**). Among these, *n* = 40 proteins comprised novel outcome predictors following severe TBI, of which an excerpt of proteins with different features are shown (**c**, **d**). These analyses were multivariable, meaning that outcome predictors are independently significant even when adjusting for previously known prognostic covariates following a severe TBI. Validation of results were conducted in an independent TBI cohort without CSF samples. Following TBI, many of the matched cohort outcome proteins were upregulated in this validation cohort as well (**e**). *CSF* cerebrospinal fluid, *MFI* median fluorescence intensity, *TBI* traumatic brain injury. All full protein names are given in Additional file 3: Table S1

There was an overlap between proteins that were significantly altered (in either CSF or serum) following TBI *and* that were altered in the CSF cluster analysis among *Q*_A_ associated proteins (Fig. 4a, b). For these we performed outcome analysis (Additional file 3: Table S5). In total, *n* = 40 proteins comprised independent outcome predictors (Additional file 3: Table S5, the representative examples CASKIN1, and matrix metalloproteinase- (MMP-)9 are highlighted in Fig. 4c, d). Importantly, numerous of these outcome proteins were also upregulated in our validation cohort following TBI (Fig. 4e). The proteins from Additional file 3: Table S5 with highest ΔNagelkerke’s pseudo-R^2^ are summarized in Table 3.

**Table 3:** BBB correlated proteins improved outcome prediction independently following severe TBI

Protein, antibody	Compartment	Highest tissue enrichment	Protein size [kDa]	Coefficient	Δ*R*^2^	adjusted *p* value	*Q*_A_ subgroup analysis
STMN4 HPA078407	CSF	cns	19.4–30.4^a^	− 0.00505	0.121	0.04548	No
C5 HPA075945	CSF	Liver/gallbladder	188.3	− 0.00095	0.106	0.04548	No
GPR26 HPA062736	CSF	cns	37.6	− 0.00684	0.099	0.04548	No
CFB HPA001817	Serum	Liver/gallbladder	85.5	0.00098	0.092	0.04548	Yes
FCN1 HPA001295	Serum	Blood	35.1	0.00303	0.082	0.04548	Yes
C9 HPA070709	CSF	Liver/gallbladder	63.2	− 0.00123	0.074	0.04548	Yes
IL6 HPA064428	Serum	Adipose/soft tissue	13.9–23.7^a^	0.00185	0.071	0.04548	Yes

All proteins that comprised the intersect between CSF-altered proteins and CSF cluster-derived proteins or serum-altered proteins and CSF cluster derived proteins were used for outcome analysis. Outcome prediction was conducted by univariable followed by multivariable proportional odds regression analysis where GOS was used as dependent variable and the protein level as independent variable. The IMPACT variables were used as covariates. Here we show the *n* = 3 proteins that conferred the highest ΔNagelkerke’s pseudo-R^2^ (decimal number) in CSF (row 1–3), in serum (row 4, 5, 7), and upon specific outcome analysis for proteins significantly different between patients with intact and disrupted BBB (row 4–6). Proteins that were significantly different between disrupted and intact BBB (CFB, FCN1, C9, IL-6) were subjected to a sub-group analysis (“*Q*_A_ subgroup analysis” column), for which adjusted *p* values are described in Additional file 3: Table S7. Protein sizes were derived from the Human Protein Atlas for the specific protein splice variant represented by the Human Protein Atlas antibody

*BBB* blood–brain barrier injury, *CNS* central nervous system, *Coeff*. regression coefficient, *CSF* cerebrospinal fluid, *Da* Dalton, *GOS* Glasgow Outcome Score, *IMPACT* International Mission for Prognosis and Analysis of Clinical Trials in TBI, *TBI* traumatic brain injury, *Q*_*A*_ albumin quotient. All full protein names are listed in Additional file 3: Table S1

^a^Protein size interval provided as Human Protein Atlas antibody is not specified for protein splice variant

We also analyzed our proteins against the dichotomized GOS, for which no proteins were significant. As this might have been caused by a type II error due to the loss of power associated with ordinal variable dichotomization [70], we re-did this analysis on imputed data (Additional file 3: Table S6). Both levels of structural proteins (e.g. MBP, p_imputed_ = 0.002), and inflammatory proteins (e.g. C9, p_imputed_ = 0.034) in CSF were predictive of outcome. We also conducted outcome analyses for the proteins significantly different between patients with intact and disrupted BBB (Additional file 3: Table S7). Among proteins that had significantly altered levels if the TBI patient had a BBB injury we found independent outcome predictors (Additional file 3: Table S7, Table 3). For these, we conducted a step-down analysis, comprising all proteins significant within the specific compartment upon multivariable analysis followed by sequential deletion until merely significant proteins were retained in the model. C9 (*p* = 0.0143, Δ*R*^2^ = 7.4%) was the only protein retained in CSF and CFB (*p* = 0.0031, Δ*R*^2^ = 9.2%) the only protein in serum.

## Discussion

We conducted a prospective, proteomic study of 177 proteins analyzed in matched CSF and serum samples of 90 severe TBI patients and 15 control subjects. Being one of the largest proteomic studies yet conduced following severe TBI, it allows us to define protein pathway alterations in CSF and serum in parallel. Specifically, we analyzed neuroinflammatory protein alterations in relation to BBB disruption, two key secondary injuries following TBI. We show that BBB disruption is an important outcome predictor following TBI, and that a protein signature comprised of predominantly neuroinflammatory pathways in CSF coincide with BBB disruption, while also serving as novel proteins of clinical importance for prognosis.

### A novel approach in TBI studies: targeting secondary injury mechanisms in large patient cohorts

We analyzed proteins of relevance for BBB disruption, a key TBI secondary injury for which there is currently no treatment [4, 71]. We utilized an antibody array [28], enabling multiplexing across a large range of protein concentrations, with low measurement variability [29]. We included a larger patient cohort than previous proteomic studies in TBI [18–22, 24–26, 72–74], thus enabling outcome analyses and APOE genotyping. Two pediatric TBI studies on smaller patient cohorts [73, 74] and one study on adult TBI patients [75] have employed similar approaches, albeit with methodological restrictions that precluded analysis of the relationship between BBB disruption and neuroinflammation, which we managed by concurrent serum and CSF sampling. We thus provide a novel framework for secondary injury studies following TBI.

### TBI studies benefit from CSF, but warrant a new BBB disruption metric

We found important differences in protein composition between the two compartments CSF and serum, within which patients grouped depending on diagnosis and BBB integrity. This was more evident in CSF than in serum, indicating that CSF might confer pivotal pathophysiological information in TBI studies. Our approach enabled quantification of BBB disruption and we found that 32% of our TBI patients suffered a BBB injury, using *Q*_A_. This is unexpectedly low in a severe TBI cohort. We hypothesize that albumin was possibly washed-out from CSF, as samples were in median obtained around 2 days following the trauma, a time-frame during which the acute vasogenic edema has been shown to be mixed with a concurrent cytotoxic edema and a delayed vasogenic edema has not yet occurred [3]. This highlights that *Q*_A_ might be suboptimal to use as a BBB integrity metric following TBI. Yet, we could show that *Q*_A_ in itself was a strong outcome predictor. Taken together, CSF is key for proteomic studies following TBI and important injury features might be accidentally surpassed if exclusively considering blood. Further, even though *Q*_A_ is the current golden-standard method for BBB integrity, the TBI field would benefit from a new BBB integrity biomarker. Radiological tools, notably dynamic enhanced contrast magnetic resonance imaging, have been utilized in other neurological disorders to assess BBB disruption [9], but has as of yet been but sparsely utilized in the TBI setting [76, 77]. Meanwhile, we show that BBB disruption measured utilizing *Q*_A_ is a novel important outcome predictor following severe TBI.

### Structural proteins altered following TBI and BBB disruption reflect pathophysiologically relevant biomarkers

We confirmed protein alterations of current TBI biomarkers as well as protein and pathway alterations of proteins less studied following TBI. The proteins MBP and AQP4 were both increased following TBI. Unlike previous biomarkers, MBP has an oligodendrocytic origin and is a tentative TBI biomarker in the post-acute phase [78]. AQP4 is an astrocytic protein, distributed along  astrocytic end feet lining the BBB [79], thus presumably reflecting structural BBB pathophysiology in our material. Previous experimental work has shown that AQP4 is globally increased following TBI, but with a decreased perivascular expression pattern [80] in line with our findings. AQP4 has also been implicated in edema development and resolution [81]. We also found upregulation of two structural protein pathways. First, we found the “synaptic proteins at the synaptic junction” pathway, entailing the spectrin proteins SPTAN1 and SPTBN1. The breakdown product of these proteins have been implicated in calpain- and caspase-mediated proteolysis and shown to be related to prognosis [82]. We also found the pathway “hypoxia-inducible factor in the cardiovascular system”, and in concordance [83] the proteins HIF1A, VEGFA, and LDHA to be upregulated, speculatively related to metabolic dysfunction. In summary, while corroborating earlier data on some of the previously known TBI biomarkers, we also provide data on novel structural proteins, which possibly reflects ongoing pathophysiology within the CNS and hence a valuable addition to the TBI biomarker literature.

### TBI and BBB disruption yields an innate immune response with marked increase of complement proteins

In both CSF and serum, TBI upregulated innate immune system pathways, which were also upregulated in CSF following BBB disruption. BBB disruption is intertwined with neuroinflammation [7], commencing when blood-borne factors leak across the disrupted BBB and tissue injury-mediated release of alarmins trigger CNS innate immune mechanisms [71, 84, 85]. This yields microglial- and inflammasome-mediated production of the cytokines IL1-β, IL-6, TNF-α, and IL-18 [84]. Both IL1-β and TNF-α can further increase BBB permeability [71]. Moreover, microglia-mediated production of IL-1α, TNFα, and C1q was recently shown to activate astrocytes [86], known to respond by IL-6 and MMP-9 production. Both IL-1α, IL-1β, IL-6, and MMP-9 were increased following TBI in our material. MMP-9 stimulates BBB disruption through degradation of tight junction and extracellular matrix proteins, while also triggering further neuroinflammation [87]. IL-6 has been suggested to be intertwined with TGF-β [12], one of the upregulated pathways that we observed. Previously, TGF-β has been shown to be increased following TBI, correlate with, and even cause BBB disruption [12, 88]. Finally, across all our comparisons complement pathways, a key element within the neuroinflammatory response [89], were implicated. Panels of elevated complement proteins have been found in blood [90], CSF [13, 18, 20, 91], and brain parenchyma [24, 42, 92] following TBI. As we assessed all complement pathways, we can corroborate many of these findings. Complement activation following TBI has been shown to occur both through systemic complement leakage across the disrupted BBB and through local CNS complement activation [93]. In line with this, and earlier data [13, 14], complement CSF/serum ratios were highly correlated with *Q*_A_. Among TBI patients with intact or disrupted BBB, a handful of primarily complement proteins were altered in CSF, congruent with descriptions that complement activation might aggravate BBB disruption [93]. We did not find any relationship between APOE genotype and our proteins. Although important, this finding should be cautiously interpreted as few TBI patients were homozygotes for APOE4. In summary, BBB disruption and neuroinflammation following TBI mutually stimulate and aggravate one another, which in our material can be quantitatively assessed in a more comprehensive fashion than before.

### Altered proteins comprise novel predictors of long-term functional outcome

One application of our findings is to use structural proteins as markers of damaged parenchyma/BBB, and neuroinflammatory proteins as novel treatment targets. In total, we found 40 predominantly CNS enriched or neuroinflammatory proteins that comprised novel, independent outcome predictors following severe TBI. Individually, these proteins explained ~ 10% additional model variance, demonstrating that a large amount of unexplained variation in TBI outcome emanates from secondary injuries. The protein with highest additional variance was STMN4 in CSF, belonging to a protein family with microtubule-destabilizing capacity [94] but also of importance for neuronal regeneration [95]. We hypothesize that STMN4 in this context serves as a metric for CNS cell death. Other proteins with high amount of additionally explained variance were neuroinflammatory proteins, notably from the complement system. Among proteins significantly different between patients with and without disrupted BBB, CFB and C9 were unique outcome predictors. Experimental TBI studies have linked variations in complement activation to worsened functional outcome [96]. Knock-out and complement inhibition models have improved outcome [92, 97–99], whereas inhibition of complement inhibition has worsened it [100]. Recently, membrane-attack complex inhibition was shown to attenuate acute TBI deficits, whereas complement protein C3 inhibition was needed to improve long-term outcome. Overall, the alternative pathway was implicated as key following TBI [101]. We cannot draw as extensive conclusions, but we note that several different complement pathway proteins comprised outcome predictors, indicating that a common therapeutic target is of interest for future studies. We thus link for the first-time proteomic data with BBB disruption, neuroinflammation, and clinical outcome within one TBI study.

### Limitations

Several limitations must be acknowledged. The supervised protein selection, although hypothesis-driven, is biased by definition. Still, as the TBI literature on unbiased approaches is vast there is a need for secondary injury mechanism focused studies on larger patient cohorts, such as ours. Further, our study is limited to cross-sectional data, which is problematic as our sampling was not entirely synchronized between or even within patients. This might cause us to miss important longitudinal protein alterations, known to be time-sensitive from preclinical research [84]. In contrast, our current findings become even more robust, as they manifest in spite of less stringent sampling. Other limitations concern discrepancies between the TBI and control subjects. Controls were younger than the TBI patients, thus possibly exaggerating the observed protein differences. Yet, they were healthy, which we considered superior compared with utilizing other patient groups with EVD/shunt treatment. Further, CSF was obtained through an EVD among TBI patients and through lumbar puncture among control subjects. An EVD decreases the external validity of the study, as patients for ethical reasons cannot be randomized to EVD treatment and an EVD would not be ethical to insert in healthy controls. This warrants for caution in CSF proteome comparisons, as CSF protein content varies along the rostro-caudal axis [68, 78]. Moreover, CSF protein levels could fail to portray intracellular alterations [19]. For this, one would need brain tissue biopsies, difficult to obtain in larger-scale quantities. Moreover, a small biopsy cannot confer global information on protein alterations within the CNS [19]. The similar limitation holds true for microdialysis [102]. Hence, CSF constitutes the state-of-the-art matrix within TBI studies of global CNS markers [18]. For us, CSF was therefore the superior biofluid to use, but future, external validation on a smaller protein-panel ought to be conducted using microdialysis as has been done in other studies [23, 103].

## Conclusion

We have examined the interplay between BBB disruption and neuroinflammation that commonly ensue a severe TBI. We have found that neuroinflammatory processes are intimately linked with BBB disruption and that both BBB disruption and numerous neuroinflammatory proteins serve as novel outcome predictors, adding ~ 10% additional variance to TBI outcome prediction models, suggesting that future efforts should strive to develop therapeutic targets towards these secondary injuries.

## Supplementary Information

**Additional file 1: Supplementary Figure 1 (Figure S1). **

**Additional file 2: Supplementary Figure 2 (Figure S2).**

**Additional file 3: Supplementary Tables 1–7 (Tables S1–S7).**

**Additional file 4: Supplementary methods, supplementary results, supplementary figure legends.**

**Additional file 5: Supplementary Figure 3 (Figure S3). **

**Additional file 6: Supplementary Figure 4 (Figure S4). **

**Additional file 7: Supplementary Figure 5 (Figure S5).**

**Additional file 8: Supplementary Figure 6 (Figure S6). **

**Additional file 9: Supplementary Figure 7 (Figure S7).**

**Additional file 10: Supplementary Figure 8 (Figure S8).**

**Additional file 11: Supplementary Figure 9 (Figure S9).**

## Abbreviations

AIS: Abbreviated Injury Scale
APOE: Apolipoprotein E
APOE4: Apolipoprotein E epsilon 4-allele
BBB: Blood–brain barrier
BSA: Bovine serum albumin
CNS: Central nervous system
CSF: Cerebrospinal fluid
CT: Computerized tomography
EDC: 1-Ethyl-3-(3-dimethylaminopropyl)carbodiimide hydrochloride
EDTA: Ethylenediaminetetraacetic acid
ELISA: Enzyme-linked immunosorbent assay
EVD: External ventricular drain
FDR: False-discovery rate
FP-TDI: Fluorescent polarization template dye incorporation
GFAP: Glial fibrillary acidic protein
GCS: Glasgow Coma Scale
GOS: Glasgow Outcome Score
HPA: Human protein atlas
ICP: Intracranial pressure
IMPACT: International Mission for Prognosis and Clinical Trial
ISS: Injury Severity Score
IQR: Interquartile range
MBP: Myelin basic protein
MES: 2-(*N*-Morpholino)ethanesulfonic acid
MFI: Median fluorescence intensity
MMP: Matrix metalloproteinase
NCCU: Neuro-critical care unit
NF-κB: Nuclear factor kappa-light-chain-enhancer of activated B cells
NFL: Neurofilament-light
NSE: Neuron-specific enolase
NX: Normalized expression
PBS: Phosphate-buffered saline
PBS-T: Phosphate-buffered saline with Tween 20
*Q*_A_: Albumin quotient
SNP: Single-nucleotide polymorphism
sulfo-NHS: N-hydroxysulfosuccinimide
TBI: Traumatic brain injury
t-SNE: T-distributed stochastic neighbor embedding
UCHL1: Ubiquitin carboxy-terminal hydrolase-L1

## Availability of data and materials

The datasets used and/or analysed during the current study are available from the corresponding author on reasonable request in a format that adheres to current Swedish and European Union legislation regarding study participant anonymity.

## References

[1] Hyder AA, Wunderlich CA, Puvanachandra P, Gururaj G, Kobusingye OC (2007). The impact of traumatic brain injuries: a global perspective. NeuroRehabilitation.

[2] Werner C, Engelhard K (2007). Pathophysiology of traumatic brain injury. Br J Anaesth.

[3] Lemarchant S, Badaut J, Plesnila N, Badaut J (2017). Brain edema formation in traumatic brain injury. Brain edema: from molecular mechanisms to clinical practice.

[4] Shlosberg D, Benifla M, Kaufer D, Friedman A (2010). Blood–brain barrier breakdown as a therapeutic target in traumatic brain injury. Nat Rev Neurol.

[5] Schwarzmaier SM, Zimmermann R, McGarry NB, Trabold R, Kim SW, Plesnila N (2013). In vivo temporal and spatial profile of leukocyte adhesion and migration after experimental traumatic brain injury in mice. J Neuroinflamm.

[6] Turtzo LC, Lescher J, Janes L, Dean DD, Budde MD, Frank JA (2014). Macrophagic and microglial responses after focal traumatic brain injury in the female rat. J Neuroinflamm.

[7] Jassam YN, Izzy S, Whalen M, McGavern DB, El Khoury J (2017). Neuroimmunology of traumatic brain injury: time for a paradigm shift. Neuron.

[8] Montagne A, Nation DA, Sagare AP, Barisano G, Sweeney MD, Chakhoyan A (2020). APOE4 leads to blood–brain barrier dysfunction predicting cognitive decline. Nature.

[9] Heye AK, Culling RD, Valdés Hernández MDC, Thrippleton MJ, Wardlaw JM (2014). Assessment of blood-brain barrier disruption using dynamic contrast-enhanced MRI. A systematic review. NeuroImage Clin.

[10] Tibbling G, Link H, Ohman S (1977). Principles of albumin and IgG analyses in neurological disorders. I. Establishment of reference values. Scand J Clin Lab Investig.

[11] Lindblad C, Nelson DW, Zeiler FA, Ercole A, Ghatan PH, von Horn H (2020). Influence of blood–brain barrier integrity on brain protein biomarker clearance in severe traumatic brain injury: a longitudinal prospective study. J Neurotrauma.

[12] Morganti-Kossmann MC, Hans VHJ, Lenzlinger PM, Dubs R, Ludwig E, Trentz O (1999). TGF-β Is elevated in the CSF of patients with severe traumatic brain injuries and parallels blood–brain barrier function. J Neurotrauma.

[13] Stahel PF, Trentz O, Kossmann T, Morganti-Kossmann MC, Perez D, Redaelli C (2001). Intrathecal levels of complement-derived soluble membrane attack complex (sc5b-9) correlate with blood-brain barrier dysfunction in patients with traumatic brain injury. J Neurotrauma.

[14] Bellander BM, Olafsson IH, Ghatan PH, Bro Skejo HP, Hansson LO, Wanecek M (2011). Secondary insults following traumatic brain injury enhance complement activation in the human brain and release of the tissue damage marker S100B. Acta Neurochir.

[15] Wang KKW, Ottens AK, Liu MC, Lewis SB, Meegan C, Oli MW (2005). Proteomic identification of biomarkers of traumatic brain injury. Expert Rev Proteom.

[16] Martinez BI, Stabenfeldt SE (2019). Current trends in biomarker discovery and analysis tools for traumatic brain injury. J Biol Eng.

[17] Kingsmore SF (2006). Multiplexed protein measurement: technologies and applications of protein and antibody arrays. Nat Rev Drug Discov.

[18] Sjödin MOD, Bergquist J, Wetterhall M (2010). Mining ventricular cerebrospinal fluid from patients with traumatic brain injury using hexapeptide ligand libraries to search for trauma biomarkers. J Chromatogr B Anal Technol Biomed Life Sci.

[19] Abu Hamdeh S, Shevchenko G, Mi J, Musunuri S, Bergquist J, Marklund N (2018). Proteomic differences between focal and diffuse traumatic brain injury in human brain tissue. Sci Rep.

[20] Hanrieder J, Wetterhall M, Enblad P, Hillered L, Bergquist J (2009). Temporally resolved differential proteomic analysis of human ventricular CSF for monitoring traumatic brain injury biomarker candidates. J Neurosci Methods.

[21] Connor DE, Chaitanya GV, Chittiboina P, McCarthy P, Scott LK, Schrott L (2017). Variations in the cerebrospinal fluid proteome following traumatic brain injury and subarachnoid hemorrhage. Pathophysiology.

[22] Conti A, Sanchez-Ruiz Y, Bachi A, Beretta L, Grandi E, Beltramo M (2004). Proteome study of human cerebrospinal fluid following traumatic brain injury indicates fibrin(ogen) degradation products as trauma-associated markers. J Neurotrauma.

[23] Orešič M, Posti JP, Kamstrup-Nielsen MH, Takala RSK, Lingsma HF, Mattila I (2016). Human serum metabolites associate with severity and patient outcomes in traumatic brain injury. EBioMedicine.

[24] Xu B, Tian R, Wang X, Zhan S, Wang R, Guo Y (2016). Protein profile changes in the frontotemporal lobes in human severe traumatic brain injury. Brain Res.

[25] Halford J, Shen S, Itamura K, Levine J, Chong AC, Czerwieniec G (2017). New astroglial injury-defined biomarkers for neurotrauma assessment. J Cereb Blood Flow Metab.

[26] Harish G, Mahadevan A, Pruthi N, Sreenivasamurthy SK, Puttamallesh VN, Keshava Prasad TS (2015). Characterization of traumatic brain injury in human brains reveals distinct cellular and molecular changes in contusion and pericontusion. J Neurochem.

[27] Pin E, Sjöberg R, Andersson E, Hellström C, Olofsson J, Jernbom Falk A, Santamaría E, Fernández-Irigoyen J (2019). Array-based profiling of proteins and autoantibody repertoires in CSF. Cerebrospinal fluid (CSF) proteomics: methods and protocols.

[28] Schwenk JM, Gry M, Rimini R, Uhlén M, Nilsson P (2008). Antibody suspension bead arrays within serum proteomics. J Proteome Res.

[29] Schwenk JM, Nilsson P, Wu CJ (2011). Antibody suspension bead arrays. Protein microarray for disease analysis methods and protocols.

[30] Cohen J (1988). Statistical power analysis for the behavioral sciences.

[31] Lakens D (2013). Calculating and reporting effect sizes to facilitate cumulative science: a practical primer for t-tests and ANOVAs. Front Psychol.

[32] Champely S. pwr: Basic functions for power analysis. version 1. R package; 2018.

[33] Thelin EP, Johannesson L, Nelson D, Bellander BM (2013). S100B is an important outcome predictor in traumatic brain injury. J Neurotrauma.

[34] Carney N, Totten AM, O’Reilly C, Ullman JS, Hawryluk GWJ, Bell MJ (2017). Guidelines for the management of severe traumatic brain injury, fourth edition. Neurosurgery.

[35] Karolinska University Hospital Laboratory. Albuminkvot, Csv/S- [Internet]. Stockholm: Klinisk kemi och KUL 24Sju. https://www.karolinska.se/KUL/Alla-anvisningar/Anvisning/9993.

[36] Isung J. Neuroinflammatory biomarkers in suicidal behavior [dissertation on the Internet]. Stockholm: Karolinska Institutet; 2016. Cited 28 Sept 2020.

[37] Thelin EP, Al Nimer F, Frostell A, Zetterberg H, Blennow K, Nystrom H (2019). A serum protein biomarker panel improves outcome prediction in human traumatic brain injury. J Neurotrauma.

[38] Chen X, Levine L, Kwok PY (1999). Fluorescence polarization in homogeneous nucleic acid analysis. Genome Res.

[39] Neiman Kungliga Tekniska Högskolan M. Bead based protein profiling in blood.

[40] Sjostedt E, Fagerberg L, Hallstrom BM, Haggmark A, Mitsios N, Nilsson P (2015). Defining the human brain proteome using transcriptomics and antibody-based profiling with a focus on the cerebral cortex. PLoS ONE.

[41] Woodcock T, Morganti-Kossmann MC (2013). The role of markers of inflammation in traumatic brain injury. Front Neurol.

[42] Bellander B-M, Singhrao SK, Ohlsson M, Mattsson P, Svensson M (2001). Complement activation in the human brain after traumatic head injury. J Neurotrauma.

[43] Helmy A, Carpenter KLH, Menon DK, Pickard JD, Hutchinson PJA (2011). The cytokine response to human traumatic brain injury: temporal profiles and evidence for cerebral parenchymal production. J Cereb Blood Flow Metab.

[44] Thelin EP, Just D, Frostell A, Häggmark-Månberg A, Risling M, Svensson M (2018). Protein profiling in serum after traumatic brain injury in rats reveals potential injury markers. Behav Brain Res.

[45] Salim A, Hadjizacharia P, Brown C, Inaba K, Teixeira PGR, Chan L (2008). Significance of troponin elevation after severe traumatic brain injury. J Trauma Inj Infect Crit Care.

[46] Collaborators. HPA. The Human Protein Atlas [Internet]. 2005.

[47] Drobin K, Nilsson P, Schwenk JM, Bäckvall H, Lehtiö J (2013). Highly multiplexed antibody suspension bead arrays for plasma protein profiling. Methods in molecular biology.

[48] Team RC (2018). R: A language and environment for statistical computing.

[49] Wickham H, Averick M, Bryan J, Chang W, McGowan L, François R (2019). Welcome to the Tidyverse. J Open Source Softw.

[50] Neuwirth E. RColorBrewer: ColorBrewer Palettes. 2014.

[51] Wilke CO. cowplot: Streamlined Plot Theme and Plot Annotations for “ggplot2.” Comprehensive R Archive Network (CRAN); 2019.

[52] Auguie B. gridExtra: miscellaneous functions for “grid” graphics. Comprehensive R Archive Network (CRAN); 2017.

[53] Holm S (1979). A simple sequentially rejective multiple test procedure a simple sequentially rejective multiple test procedure. Scand J Stat.

[54] Benjamini Y, Hochberg Y (1995). Controlling the false discovery rate—a practical and powerful approach to multiple testing. J R Stat Soc Ser B-Methodol.

[55] van Buuren S, Groothuis-Oudshoorn K (2011). mice: multivariate imputation by chained equations in R. J Stat Softw.

[56] Uhlen M, Fagerberg L, Hallstrom BM, Lindskog C, Oksvold P, Mardinoglu A (2015). Proteomics. Tissue-based map of the human proteome. Science (80-).

[57] The human brain—The Human Protein Atlas [Internet]. https://www.proteinatlas.org/humanproteome/brain. Cited 19 May 2020.

[58] Van Der Maaten L, Hinton G (2008). Visualizing data using t-SNE. J Mach Learn Res.

[59] Krijthe JH. Rtsne: T-distributed stochastic neighbor embedding using a Barnes-Hut implementation. GitHub; 2015.

[60] Gu Z, Eils R, Schlesner M (2016). Complex heatmaps reveal patterns and correlations in multidimensional genomic data. Bioinformatics.

[61] Ulgen E, Ozisik O, Sezerman OU (2019). PathfindR: an R package for comprehensive identification of enriched pathways in omics data through active subnetworks. Front Genet.

[62] Step-by-Step Execution of the pathfindR Enrichment Workflow [Internet]. https://cran.r-project.org/web/packages/pathfindR/vignettes/manual_execution.html. Cited 30 Jul 2020.

[63] Chen H. VennDiagram: generate high-resolution Venn and Euler plots. CRAN; 2018.

[64] Harrell Jr FE. rms: regression modeling strategies; 2019.

[65] Murray GD, Butcher I, McHugh GS, Lu J, Mushkudiani NA, Maas AI (2007). Multivariable prognostic analysis in traumatic brain injury: results from the IMPACT study. J Neurotrauma.

[66] Nelson DW, Nystrom H, MacCallum RM, Thornquist B, Lilja A, Bellander BM (2010). Extended analysis of early computed tomography scans of traumatic brain injured patients and relations to outcome. J Neurotrauma.

[67] Thelin EP, Nelson DW, Vehvilainen J, Nystrom H, Kivisaari R, Siironen J (2017). Evaluation of novel computerized tomography scoring systems in human traumatic brain injury: an observational, multicenter study. PLoS Med.

[68] Weisner B, Bernhardt W (1978). Protein fractions of lumbar, cisternal, and ventricular cerebrospinal fluid. J Neurol Sci.

[69] Brettschneider J, Claus A, Kassubek J, Tumani H (2005). Isolated blood-cerebrospinal fluid barrier dysfunction: prevalence and associated diseases. J Neurol.

[70] Murad H, Fleischman A, Sadetzki S, Geyer O, Freedman LS (2003). Small samples and ordered logistic regression: does it help to collapse categories of outcome?. Am Stat.

[71] Chodobski A, Zink BJ, Szmydynger-Chodobska J (2011). Blood–brain barrier pathophysiology in traumatic brain injury. Transl Stroke Res.

[72] Stefini R, Catenacci E, Piva S, Sozzani S, Valerio A, Bergomi R (2008). Chemokine detection in the cerebral tissue of patients with posttraumatic brain contusions. J Neurosurg.

[73] Buttram SDW, Wisniewski SR, Jackson EK, Adelson PD, Feldman K, Bayir H (2007). Multiplex assessment of cytokine and chemokine levels in cerebrospinal fluid following severe pediatric traumatic brain injury: effects of moderate hypothermia. J Neurotrauma.

[74] Berger RP, Taasan S, Rand A, Lokshin A, Kochanek P (2009). Multiplex assessment of serum biomarker concentrations in well-appearing children with inflicted traumatic brain injury. Pediatr Res.

[75] He XY, Dan QQ, Wang F, Li YK, Fu SJ, Zhao N (2019). Protein network analysis of the serum and their functional implication in patients subjected to traumatic brain injury. Front Neurosci.

[76] Wei XE, Wang D, Li MH, Zhang YZ, Li YH, Li WB (2011). A useful tool for the initial assessment of blood-brain barrier permeability after traumatic brain injury in rabbits: dynamic contrast-enhanced magnetic resonance imaging. J Trauma Inj Infect Crit Care.

[77] Winter C, Bell C, Whyte T, Cardinal J, Macfarlane D, Rose S (2015). Blood–brain barrier dysfunction following traumatic brain injury: correlation of K trans (DCE-MRI) and SUVR (99mTc-DTPA SPECT) but not serum S100B. Neurol Res.

[78] Zetterberg H, Blennow K (2016). Fluid biomarkers for mild traumatic brain injury and related conditions. Nat Publ Gr.

[79] Abbott NJ, Rönnbäck L, Hansson E (2006). Astrocyte-endothelial interactions at the blood–brain barrier. Nat Rev Neurosci.

[80] Ren Z, Iliff JJ, Yang L, Yang J, Chen X, Chen MJ (2013). “Hit & Run” model of closed-skull traumatic brain injury (TBI) reveals complex patterns of post-traumatic AQP4 dysregulation. J Cereb Blood Flow Metab.

[81] Fukuda AM, Badaut J (2012). Aquaporin 4: a player in cerebral edema and neuroinflammation. J Neuroinflamm.

[82] Mondello S, Robicsek SA, Gabrielli A, Brophy GM, Papa L, Tepas J (2010). αII-Spectrin breakdown products (SBDPs): diagnosis and outcome in severe traumatic brain injury patients. J Neurotrauma.

[83] Ramakrishnan S, Anand V, Roy S (2014). Vascular endothelial growth factor signaling in hypoxia and inflammation. J Neuroimmune Pharmacol.

[84] Gadani SP, Walsh JT, Lukens JR, Kipnis J (2015). Dealing with danger in the CNS: the response of the immune system to injury. Neuron.

[85] Hammad A, Westacott L, Zaben M (2018). The role of the complement system in traumatic brain injury: a review. J Neuroinflamm.

[86] Liddelow SA, Guttenplan KA, Clarke LE, Bennett FC, Bohlen CJ, Schirmer L (2017). Neurotoxic reactive astrocytes are induced by activated microglia. Nature.

[87] Abdul-Muneer PM, Pfister BJ, Haorah J, Chandra N (2016). Role of matrix metalloproteinases in the pathogenesis of traumatic brain injury. Mol Neurobiol.

[88] Shen W, Li S, Chung SH, Zhu L, Stayt J, Su T (2011). Tyrosine phosphorylation of VE-cadherin and claudin-5 is associated with TGF-β1-induced permeability of centrally derived vascular endothelium. Eur J Cell Biol.

[89] Dinet V, Petry KG, Badaut J (2019). Brain-immune interactions and neuroinflammation after traumatic brain injury. Front Neurosci.

[90] Bao W, He F, Yu L, Gao J, Meng F, Ding Y (2018). Complement cascade on severe traumatic brain injury patients at the chronic unconscious stage: implication for pathogenesis. Expert Rev Mol Diagn.

[91] Kossmann T, Stahel PF, Morganti-Kossmann MC, Jones JL, Barnum SR (1997). Elevated levels of the complement components C3 and factor B in ventricular cerebrospinal fluid of patients with traumatic brain injury. J Neuroimmunol.

[92] Longhi L, Orsini F, De Blasio D, Fumagalli S, Ortolano F, Locatelli M (2014). Mannose-binding lectin is expressed after clinical and experimental traumatic brain injury and its deletion is protective. Crit Care Med.

[93] Stahel PF, Morganti-Kossmann MC, Kossmann T (1998). The role of the complement system in traumatic brain injury. Brain Res Rev.

[94] HPA Collaborators. STMN4 protein expression summary—the Human Protein Atlas. https://www.proteinatlas.org/ENSG00000015592-STMN4. Cited 3 Aug 2020.

[95] Nakazawa T, Morii H, Tamai M, Mori N (2005). Selective upregulation of RB3/stathmin4 by ciliary neurotrophic factor following optic nerve axotomy. Brain Res.

[96] Al Nimer F, Lindblom R, Strom M, Guerreiro-Cacais AO, Parsa R, Aeinehband S (2013). Strain influences on inflammatory pathway activation, cell infiltration and complement cascade after traumatic brain injury in the rat. Brain Behav Immun.

[97] Leinhase I, Rozanski M, Harhausen D, Thurman JM, Schmidt OI, Hossini AM (2007). Inhibition of the alternative complement activation pathway in traumatic brain injury by a monoclonal anti-factor B antibody: a randomized placebo-controlled study in mice. J Neuroinflamm.

[98] Rich MC, Keene CN, Neher MD, Johnson K, Yu ZX, Ganivet A (2016). Site-targeted complement inhibition by a complement receptor 2-conjugated inhibitor (mTT30) ameliorates post-injury neuropathology in mouse brains. Neurosci Lett.

[99] Fluiter K, Opperhuizen AL, Morgan BP, Baas F, Ramaglia V (2014). Inhibition of the membrane attack complex of the complement system reduces secondary neuroaxonal loss and promotes neurologic recovery after traumatic brain injury in mice. J Immunol.

[100] Stahel PF, Flierl MA, Morgan BP, Persigehl I, Stoll C, Conrad C (2009). Absence of the complement regulatory molecule CD59a leads to exacerbated neuropathology after traumatic brain injury in mice. J Neuroinflamm.

[101] Alawieh A, Langley EF, Weber S, Adkins D, Tomlinson S (2018). Identifying the role of complement in triggering neuroinflammation after traumatic brain injury. J Neurosci.

[102] Stocchetti N, Carbonara M, Citerio G, Ercole A, Skrifvars MB, Smielewski P (2017). Severe traumatic brain injury: targeted management in the intensive care unit. Lancet Neurol.

[103] Dyhrfort P, Shen Q, Clausen F, Thulin M, Enblad P, Kamali-Moghaddam M (2019). Monitoring of protein biomarkers of inflammation in human traumatic brain injury using microdialysis and proximity extension assay technology in neurointensive care. J Neurotrauma.

