# Supplementary figures and images for: Fluid proteomics of CSF and serum reveal important neuroinflammatory proteins in blood–brain barrier disruption and outcome prediction following severe traumatic brain injury: a prospective, observational study
Source: Crit Care. 2021 Mar 12;25:103. doi: 10.1186/s13054-021-03503-x (PMC7955664; doi:10.1186/s13054-021-03503-x)

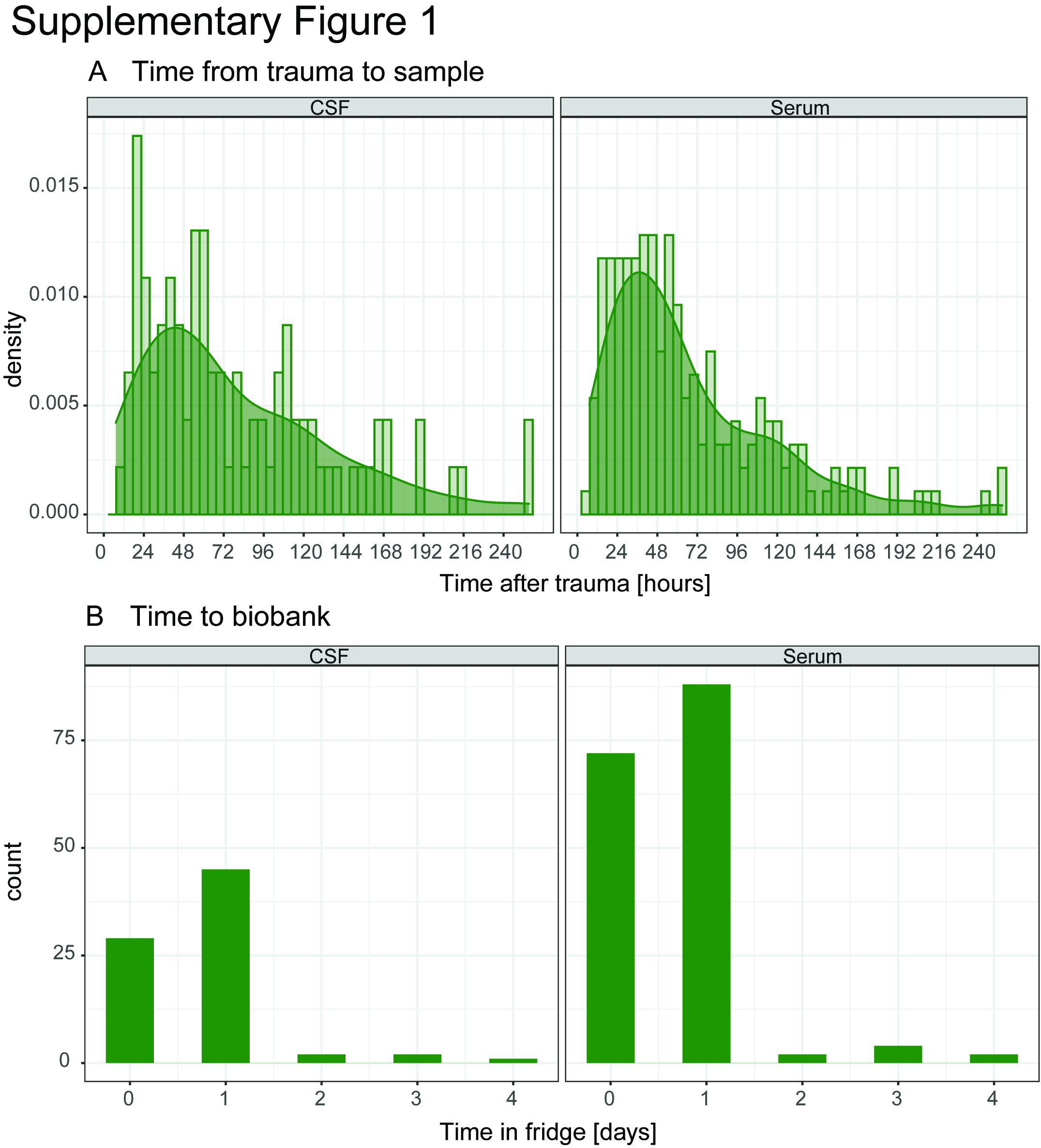

Supplement: Supplementary file 1 — Additional file 1: Supplementary Figure 1 (Figure S1). [file 13054_2021_3503_MOESM1_ESM.tif]

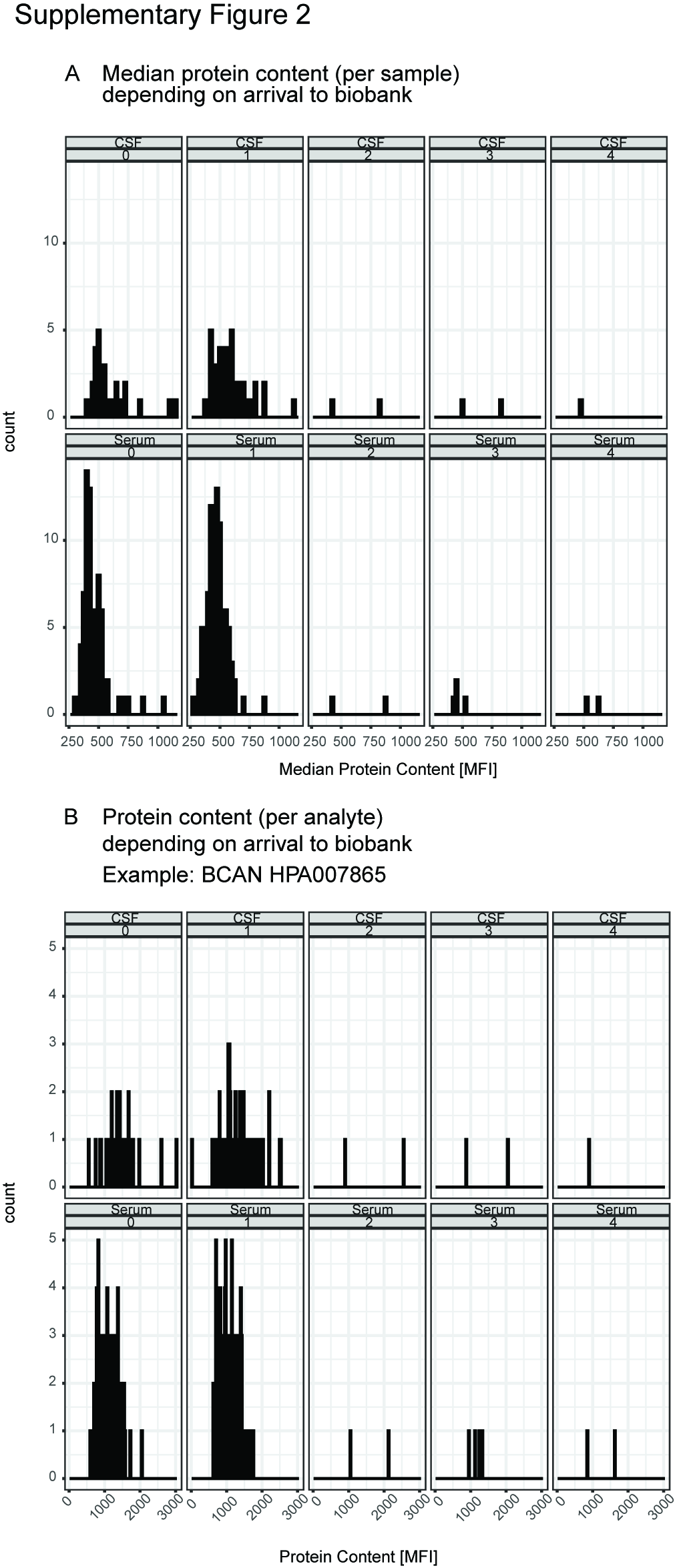

Supplement: Supplementary file 2 — Additional file 2: Supplementary Figure 2 (Figure S2). [file 13054_2021_3503_MOESM2_ESM.tif]

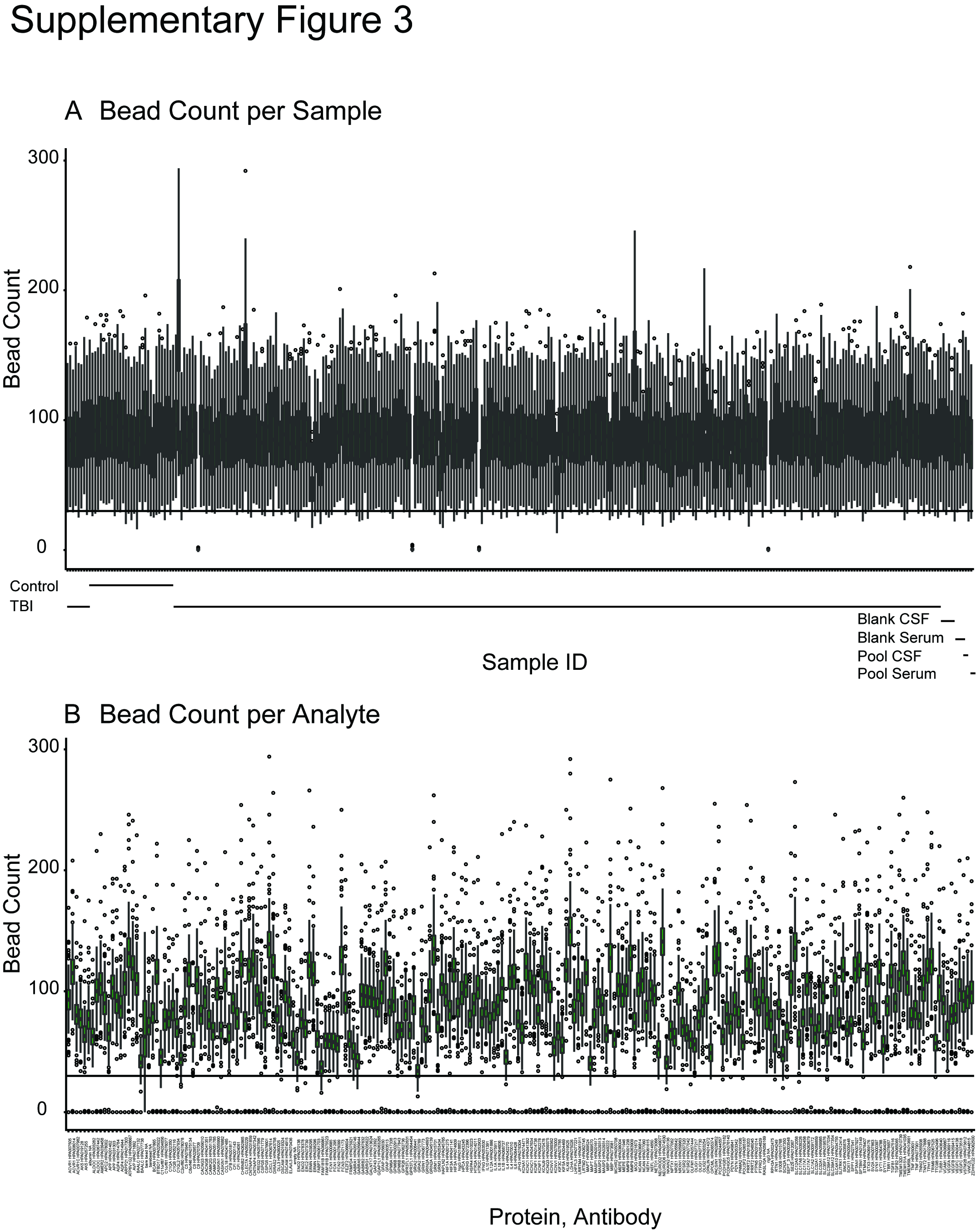

Supplement: Supplementary file 5 — Additional file 5: Supplementary Figure 3 (Figure S3). [file 13054_2021_3503_MOESM5_ESM.tif]

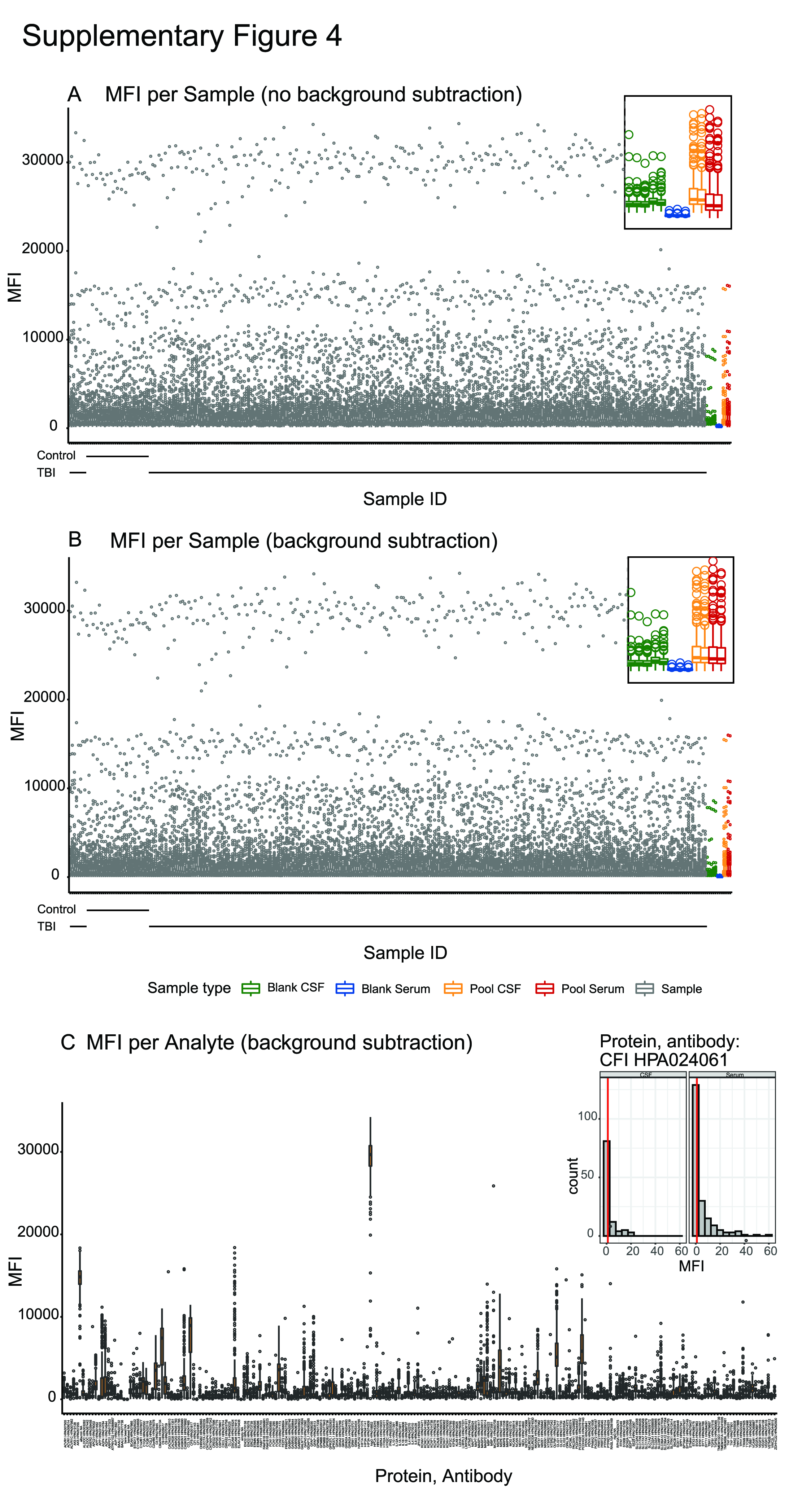

Supplement: Supplementary file 6 — Additional file 6: Supplementary Figure 4 (Figure S4). [file 13054_2021_3503_MOESM6_ESM.tif]

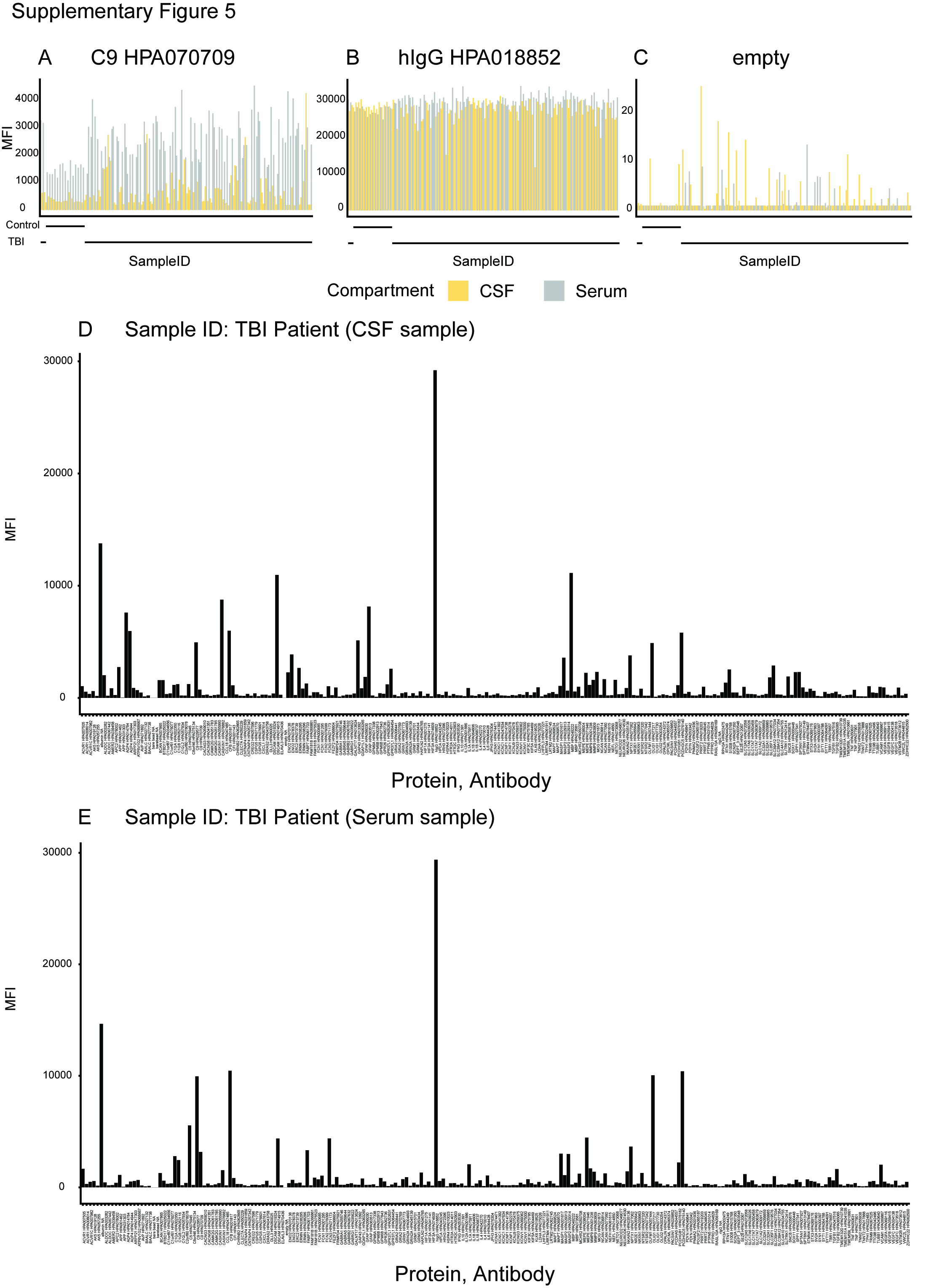

Supplement: Supplementary file 7 — Additional file 7: Supplementary Figure 5 (Figure S5). [file 13054_2021_3503_MOESM7_ESM.tif]

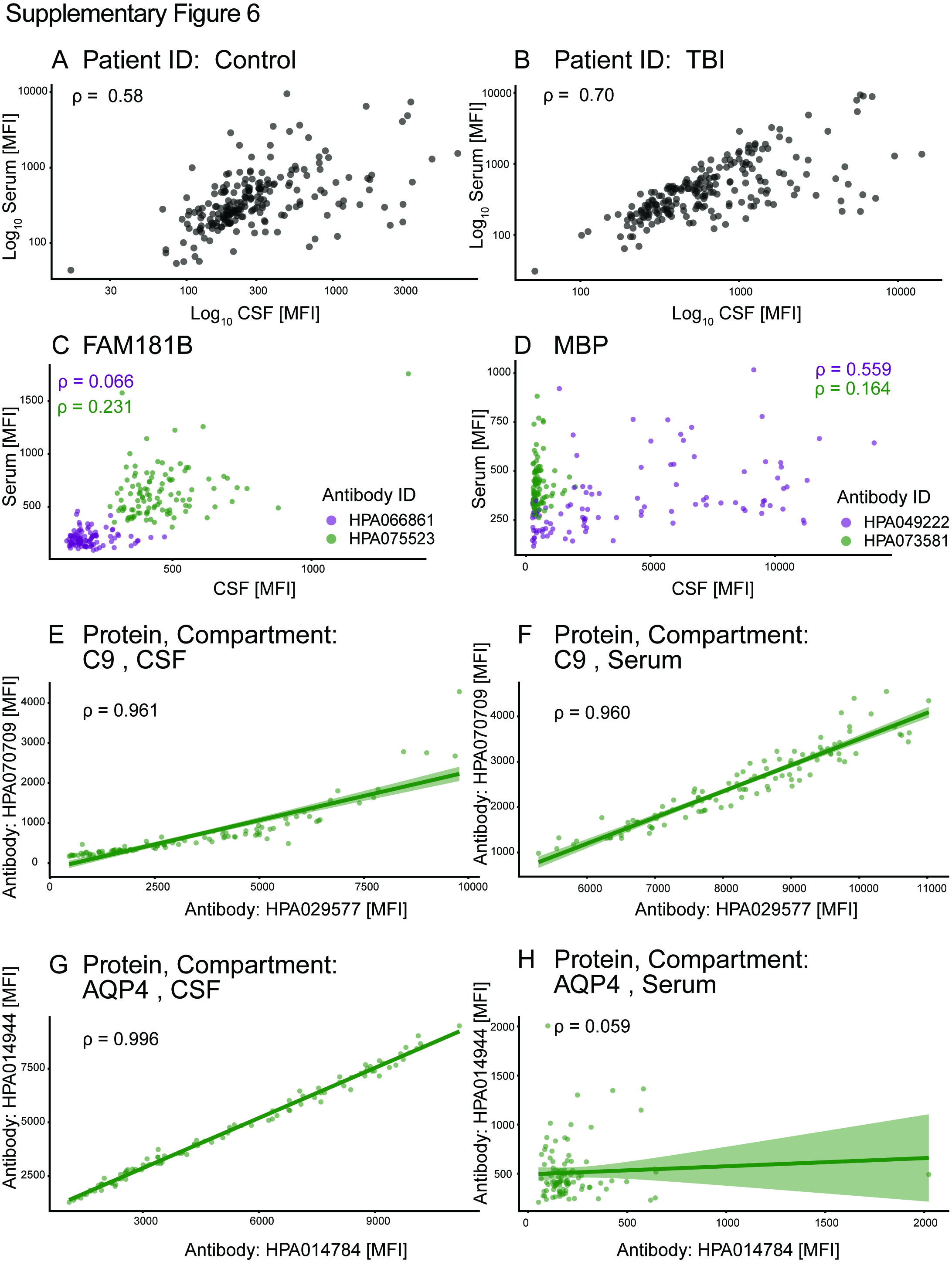

Supplement: Supplementary file 8 — Additional file 8: Supplementary Figure 6 (Figure S6). [file 13054_2021_3503_MOESM8_ESM.tif]

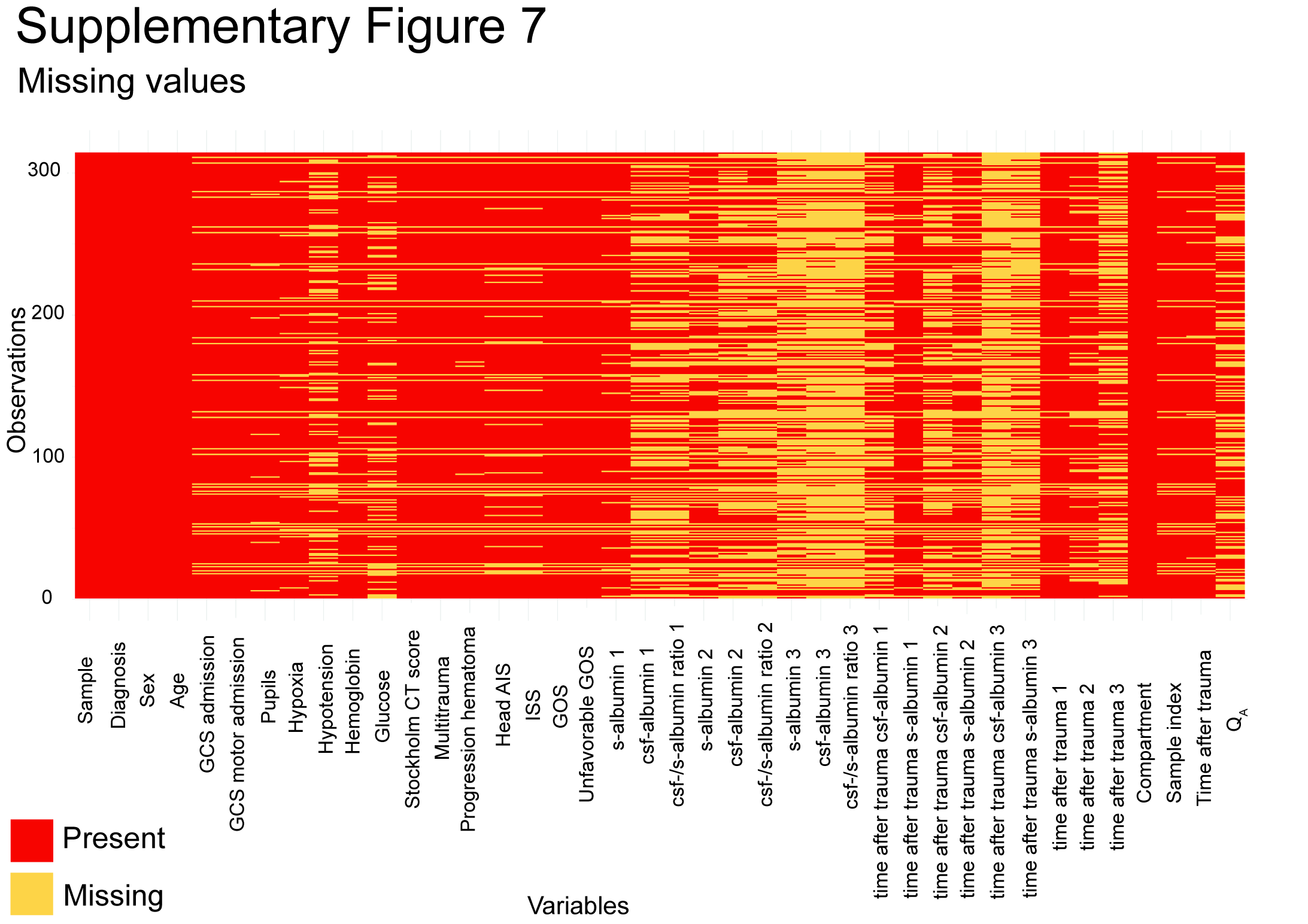

Supplement: Supplementary file 9 — Additional file 9: Supplementary Figure 7 (Figure S7). [file 13054_2021_3503_MOESM9_ESM.tif]

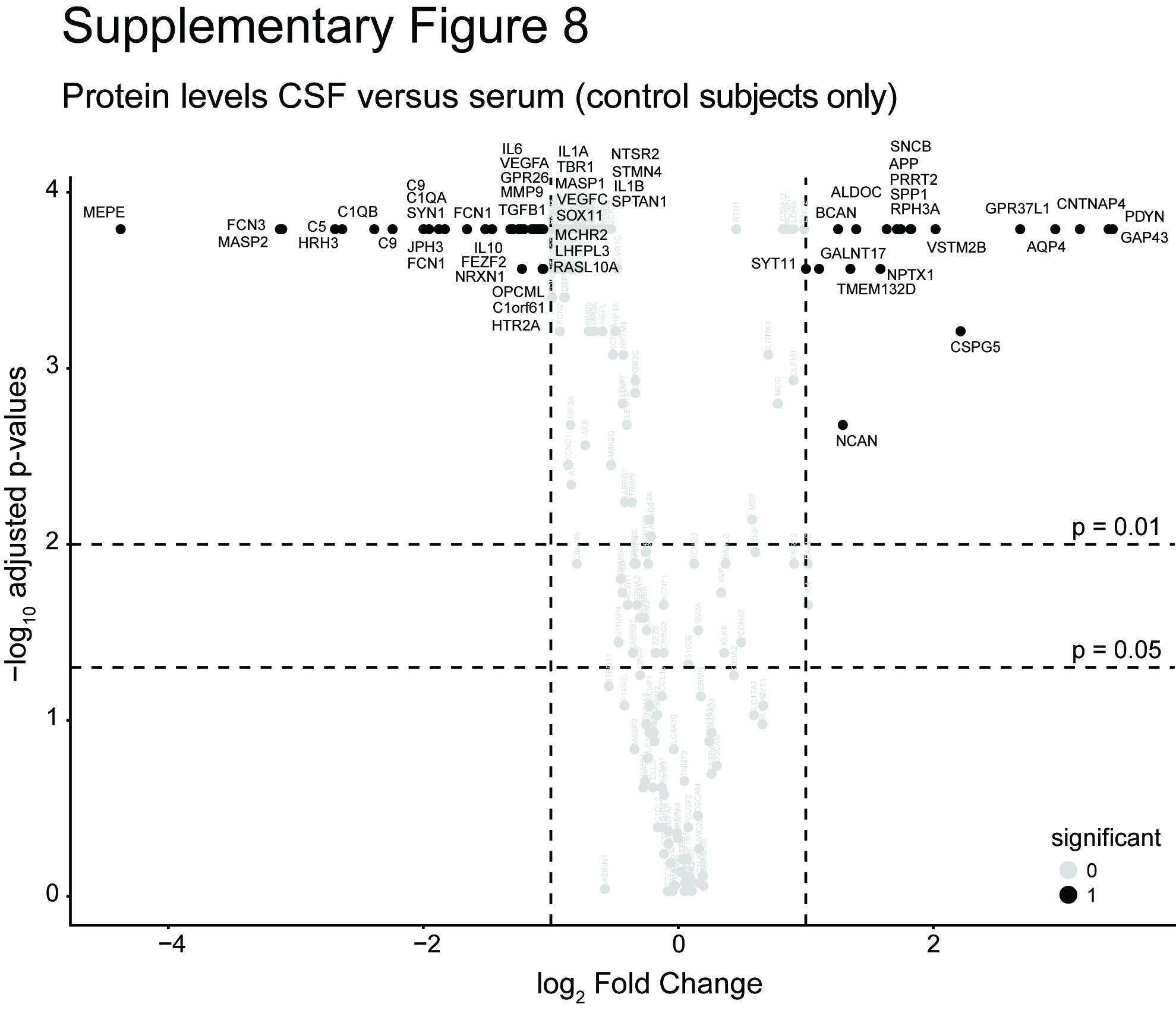

Supplement: Supplementary file 10 — Additional file 10: Supplementary Figure 8 (Figure S8). [file 13054_2021_3503_MOESM10_ESM.tif]

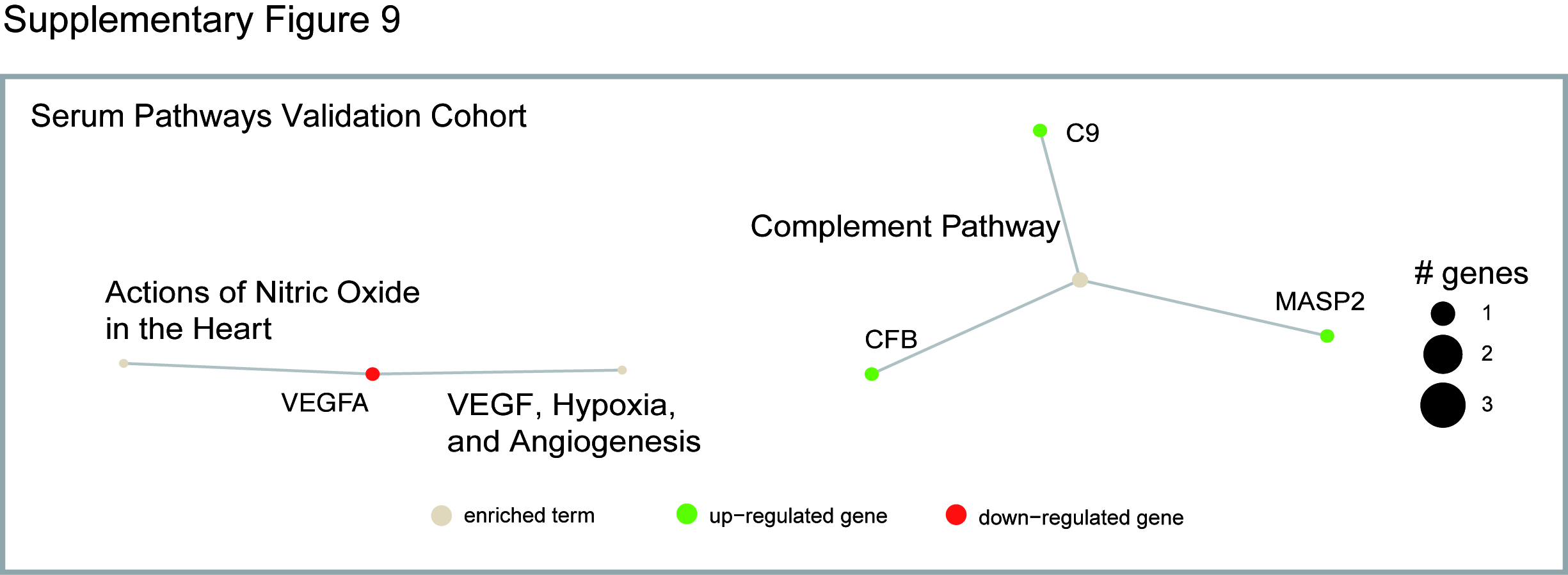

Supplement: Supplementary file 11 — Additional file 11: Supplementary Figure 9 (Figure S9). [file 13054_2021_3503_MOESM11_ESM.tif]
