# Supplementary material for: Fluid proteomics of CSF and serum reveal important neuroinflammatory proteins in blood–brain barrier disruption and outcome prediction following severe traumatic brain injury: a prospective, observational study
Source: Crit Care. 2021 Mar 12;25:103. doi: 10.1186/s13054-021-03503-x (PMC7955664; doi:10.1186/s13054-021-03503-x)
Supplement: Supplementary file 3 — Additional file 3: Supplementary Tables 1–7 (Tables S1–S7). [file 13054_2021_3503_MOESM3_ESM.docx]

| **Supplementary Table 1 (S1): List of all proteins, antibody IDs and full protein names** | | | |
| --- | --- | --- | --- |
| **Gene Name** | **Protein Name** | **UniProt ID** | **Human Protein Atlas Antibody ID** |
| ACVR1 | Activin A receptor type 1 | Q04771 | HPA007505 |
| ACVR1 | Activin A receptor type 1 | Q04771 | HPA008014 |
| ACVR1C | Activin A receptor type 1C | Q8NER5 | HPA007982 |
| AK5 | Adenylate kinase 5 | Q9Y6K8 | HPA019128 |
| AK5 | Adenylate kinase 5 | Q9Y6K8 | HPA057255 |
| ALDOC | Aldolase fructose-bisphosphate C | P09972 | HPA003282 |
| ALDOC | Aldolase fructose-bisphosphate C | P09972 | HPA067442 |
| AMER2 | APC membrane recruitment protein 2 | Q8N7J2 | HPA039458 |
| APC2 | APC2 WNT signaing pathway regulator | O95996 | HPA078002 |
| APP | Amyloid beta precursor protein | P05067 | HPA001462 |
| APP | Amyloid beta precursor protein | P05067 | HPA031303 |
| AQP4 | Aquaporin 4 | P55087 | HPA014784 |
| AQP4 | Aquaporin 4 | P55087 | HPA014944 |
| ARPP21 | CAMP regulated phosphoprotein 21 | Q9UBL0 | HPA017303 |
| ATP6V1G2 | ATPase H+ transporting V1 subunit G2 | O95670 | HPA068667 |
| AVP | Arginine vasopressin | P01185 | HPA071892 |
| BAALC | BAALC MAP3K1 and KLF4 binding | Q8WXS3 | HPA027132 |
| BAALC | BAALC MAP3K1 and KLF4 binding | Q8WXS3 | HPA077738 |
| BCAN | Brevican | Q96GW7 | HPA007865 |
| BTBD17 | BTB domain containing 17 | A6NE02 | HPA025022 |
| C11orf87 | Chromosome 11 open reading frame 87 | Q6NUJ2 | HPA034656 |
| C1orf61 | Chromosome 1 open reading frame 61 | Q13536 | HPA076077 |
| C1QA | Complement C1q A chain | P02745 | HPA002350 |
| C1QB | Complement C1q B chain | P02746 | HPA052116 |
| C1QL2 | Complement C1q like 2 | Q7Z5L3 | HPA057934 |
| C2orf80 | Chromosome 2 open reading frame 80 | Q0P641 | HPA078078 |
| C5 | Complement C5 | P01031 | HPA075945 |
| C8orf46 | Chromosome 8 open reading frame 46 | Q8TAG6 | HPA075134 |
| C9 | Complement C9 | P02748 | HPA029577 |
| C9 | Complement C9 | P02748 | HPA070709 |
| CACNG3 | Calcium voltage-gated channel auxiliary subunit gamma 3 | O60359 | HPA059010 |
| CACNG8 | Calcium voltage-gated channel auxiliary subunit gamma 8 | Q8WXS5 | HPA041351 |
| CAMK2G | Calcium/calmodulin dependent protein kinase II gamma | Q13555 | HPA051783 |
| CAMK2G | Calcium/calmodulin dependent protein kinase II gamma | Q13555 | HPA051785 |
| CASKIN1 | CASK interacting protein 1 | Q8WXD9 | HPA055990 |
| CASKIN1 | CASK interacting protein 1 | Q8WXD9 | HPA076882 |
| CCL18 | C-C motif chemokine ligand 18 | P55774 | HPA047485 |
| CFB | Complement factor B | P00751 | HPA001817 |
| CFI | Complement factor I | P05156 | HPA024061 |
| CFI | Complement factor I | P05156 | HPA001143 |
| CHRNB2 | Cholinergic receptor nicotinic beta 2 subunit | P17787 | HPA062865 |
| CLEC7A | C-type lectin domain containing 7A | Q9BXN2 | HPA050229 |
| CNTNAP4 | Contactin associated protein like 4 | Q9C0A0 | HPA031859 |
| CNTNAP4 | Contactin associated protein like 4 | Q9C0A0 | HPA057342 |
| CREG2 | Cellular repressor of E1A stimulated genes 2 | Q8IUH2 | HPA057596 |
| CSPG5 | Chondroitin sulfate proteoglycan 5 | O95196 | HPA071779 |
| CSPG5 | Chondroitin sulfate proteoglycan 5 | O95196 | HPA076601 |
| CXCL1 | C-X-C motif chemokine ligand 1 | P09341 | HPA067614 |
| DIRAS2 | DIRAS family GTPase 2 | Q96HU8 | HPA043758 |
| DLL3 | Delta like canonical Notch ligand 3 | Q9NYJ7 | HPA060025 |
| DSCAM | DS cell adhesion molecule | O60469 | HPA019324 |
| DSCAM | DS cell adhesion molecule | O60469 | HPA074915 |
| ELAVL3 | ELAV like RNA binding protein 3 | Q14576 | HPA070436 |
| ENO2 | Enolase 2 | P09104 | HPA070138 |
| ENO2 | Enolase 2 | P09104 | HPA078378 |
| ERC2 | ELKS/RAB6-interacting/CAST family member 2 | O15083 | HPA073739 |
| ERMN | Ermin | Q8TAM6 | HPA038295 |
| ERMN | Ermin | Q8TAM6 | HPA038296 |
| FABP7 | Fatty acid binding protein 7 | O15540 | HPA061703 |
| FAM181B | Family with sequence similarity 181 member B | A6NEQ2 | HPA066861 |
| FAM181B | Family with sequence similarity 181 member B | A6NEQ2 | HPA075523 |
| FCN1 | Ficolin 1 | O00602 | HPA000685 |
| FCN1 | Ficolin 1 | O00602 | HPA001295 |
| FCN2 | Ficolin 2 | Q15485 | HPA076099 |
| FCN3 | Ficolin 3 | O75636 | HPA071173 |
| FEZF2 | FEZ family zinc finger 2 | Q8TBJ5 | HPA068604 |
| FRMPD4 | FERM and PDZ domain containing 4 | Q14CM0 | HPA075921 |
| GABRA1 | Gamma-aminobutyric acid type A receptor alpha 1 subunit | P14867 | HPA055746 |
| GABRA5 | Gamma-aminobutyric acid type A receptor alpha 5 subunit | P31644 | HPA059644 |
| GABRB2 | Gamma-aminobutyric acid type A receptor beta2 subunit | P47870 | HPA067632 |
| GABRG1 | Gamma-aminobutyric acid type A receptor gamma1 subunit | Q8N1C3 | HPA035622 |
| GALNT17 | Polypeptide N-acetylgalactosaminyltransferase 17 | Q6IS24 | HPA013624 |
| GAP43 | Growth associated protein 43 | P17677 | HPA013392 |
| GDAP1L1 | Ganglioside induced differentiation associated protein 1 like 1 | Q96MZ0 | HPA063265 |
| GFAP | Glial fibrillary acidic protein | P14136 | HPA056030 |
| GFAP | Glial fibrillary acidic protein | P14136 | HPA063513 |
| GPM6A | Glycoprotein M6A | P51674 | HPA017338 |
| GPM6B | Glycoprotein M6B | Q13491 | HPA002913 |
| GPM6B | Glycoprotein M6B | Q13491 | HPA077843 |
| GPR26 | G protein-coupled receptor 26 | Q8NDV2 | HPA062736 |
| GPR37L1 | G protein-coupled receptor 37 like 1 | O60883 | HPA052631 |
| GPR37L1 | G protein-coupled receptor 37 like 1 | O60883 | HPA064454 |
| GRIA2 | Glutamate ionotropic receptor AMPA type subunit 2 | P42262 | HPA008441 |
| GRIA2 | Glutamate ionotropic receptor AMPA type subunit 2 | P42262 | HPA070769 |
| GRIN1 | Glutamate ionotropic receptor NMDA type subunit 1 | Q05586 | HPA067773 |
| GRIN2A | Glutamate ionotropic receptor NMDA type subunit 2A | Q12879 | HPA004693 |
| GRIN2A | Glutamate ionotropic receptor NMDA type subunit 2A | Q12879 | HPA045139 |
| GRM1 | Glutamate metabotropic receptor 1 | Q13255 | HPA015701 |
| GRM3 | Glutamate metabotropic receptor 3 | Q14832 | HPA053434 |
| HAPLN2 | Hyaluronan and proteoglycan link protein 2 | Q9GZV7 | HPA045765 |
| HIF1A | Hypoxia inducible factor 1 alpha subunit | Q16665 | HPA001275 |
| HIF3A | Hypoxia inducible factor 3 alpha subunit | Q9Y2N7 | HPA041141 |
| HIF3A | Hypoxia inducible factor 3 alpha subunit | Q9Y2N7 | HPA074609 |
| HPCA | Hippocalcin | P84074 | HPA043245 |
| HRH3 | Histamine receptor H3 | Q9Y5N1 | HPA072396 |
| HSPA4 | Heat shock protein family A (Hsp70) member 4 | P34932 | HPA010023 |
| HTR2A | 5-hydroxytryptamine receptor 2A | P28223 | HPA014011 |
| HTR2C | 5-hydroxytryptamine receptor 2C | P28335 | HPA052903 |
| IFNG | Interferon gamma | P01579 | HPA053530 |
| IL10 | Interleukin 10 | P22301 | HPA071391 |
| IL12A | Interleukin 12A | P29459 | HPA001886 |
| IL1A | Interleukin 1 alpha | P01583 | HPA075911 |
| IL1B | Interleukin 1 beta | P01584 | HPA064606 |
| IL1B | Interleukin 1 beta | P01584 | HPA068737 |
| IL4 | Interleukin 4 | P05112 | HPA042270 |
| IL4 | Interleukin 4 | P05112 | HPA070010 |
| IL6 | Interleukin 6 | P05231 | HPA064428 |
| JPH3 | Junctophilin 3 | Q8WXH2 | HPA076304 |
| KCNA1 | Potassium voltage-gated channel subfamily A member 1 | Q09470 | HPA074471 |
| KCNC1 | Potassium voltage-gated channel subfamily C member 1 | P48547 | HPA041392 |
| KCNC1 | Potassium voltage-gated channel subfamily C member 1 | P48547 | HPA047634 |
| KCNF1 | Potassium voltage-gated channel modfier subfamily F member 1 | Q9H3M0 | HPA014738 |
| KCNF1 | Potassium voltage-gated channel modifier subfamily F member 1 | Q9H3M0 | HPA062278 |
| KCNJ9 | Potassium voltage-gated channel subfamily J member 9 | Q92806 | HPA070478 |
| KCNQ3 | Potassium voltage-gated channel subfamily Q member 3 | O43525 | HPA059375 |
| KCNV1 | Potassium voltage-gated channel modifier subfamily V member 1 | Q6PIU1 | HPA069362 |
| KCNV1 | Potassium voltage-gated channel modifier subfamily V member 1 | Q6PIU1 | HPA075000 |
| KIF3C | Kinesin family member 3C | O14782 | HPA075785 |
| KIF5A | Kinesn family member 5A | Q12840 | HPA004469 |
| KLK6 | Kallikrein related peptidase 6 | Q92876 | HPA019525 |
| LDHA | Lactate dehydrogenase A | P00338 | HPA075026 |
| LHFPL3 | LHFPL tetraspan subfamily member 3 | Q86UP9 | HPA077221 |
| LRRTM4 | Leucine rich repeat transmembran neuronal 4 | Q86VH4 | HPA061911 |
| LRTM2 | Leucine rich repeats and transmembraine domains 2 | Q8N967 | HPA062745 |
| MAPT | Microtubule associated protein tau | P10636 | HPA069570 |
| MAPT | Microtubule associated protein tau | P10636 | HPA069524 |
| MASP1 | Mannan binding lectin serine peptidase 1 | P48740 | HPA001617 |
| MASP2 | Mannan binding lectin serine peptidase 2 | O00187 | HPA029314 |
| MASP2 | Mannan binding lectin serine peptidase 2 | O00187 | HPA029313 |
| MBP | Myelin basic protein | P02686 | HPA049222 |
| MBP | Myelin basic protein | P02686 | HPA073581 |
| MCHR2 | Melanin concentrating hormone receptor 2 | Q969V1 | HPA050708 |
| MEPE | Matrix extracellular phosphoglycoprotein | Q9NQ76 | HPA038004 |
| MEPE | Matrix extracellular phosphoglycoprotein | Q9NQ76 | HPA071946 |
| MMP9 | Matrix metallopeptidase 9 | P14780 | HPA001238 |
| MMP9 | Matrix metallopeptidase 9 | P14780 | HPA063909 |
| MOG | Myelin oligodendrocyte glycoprotein | Q16653 | HPA021873 |
| NCAN | Neurocan | O14594 | HPA036814 |
| NCAN | Neurocan | O14594 | HPA077060 |
| NEFH | Neurofilament heavy | P12036 | HPA061615 |
| NEFL | Neurofilament light | P07196 | HPA014850 |
| NETO1 | Neuropilin and tolloid like 1 | Q8TDF5 | HPA073068 |
| NEUROD2 | Neuronal differentiation 2 | Q15784 | HPA049077 |
| NEUROD6 | Neuronal differentiation 6 | Q96NK8 | HPA074530 |
| NKAIN2 | Sodium/potassium transporting ATPase interacting 2 | Q5VXU1 | HPA035136 |
| NPTX1 | Neuronal pentraxin 1 | Q15818 | HPA077062 |
| NR2E1 | Nuclear receptor subfamily 2 group E member 1 | Q9Y466 | HPA055642 |
| NRXN1 | Neurexin 1 | Q9ULB1 | HPA059963 |
| NTSR2 | Neurotensin receptor 2 | O95665 | HPA007320 |
| NTSR2 | Neurotensin receptor 2 | O95665 | HPA077042 |
| OLFM1 | Olfactomedin 1 | Q99784 | HPA057444 |
| OLIG1 | Oligdendrocyte transcription factor 1 | Q8TAK6 | HPA077217 |
| OLIG1 | Oligodendrocyte transcription factor 1 | Q8TAK6 | HPA077730 |
| OLIG2 | Oligodendrocyte transcription factor 2 | Q13516 | HPA003254 |
| OPALIN | Oligodendrocytic myelin paranodal and inner loop protein | Q96PE5 | HPA014372 |
| OPCML | Opioid binding protein/cell adhesion molecule like | Q14982 | HPA065374 |
| PACSIN1 | Protein kinase C and casein kinase substrate in neurons 1 | Q9BY11 | HPA028852 |
| PCDHA5 | Protocadherin alpha 5 | Q9Y5H7 | HPA044557 |
| PCDHGB1 | Protocadherin gamma subfamily B 1 | Q9Y5G3 | HPA076182 |
| PCDHGC5 | Protocadherin gamma subfamily C 5 | Q9Y5F6 | HPA076140 |
| PDYN | Prodynorphin | P01213 | HPA049841 |
| PDYN | Prodynorphin | P01213 | HPA053342 |
| PNMA2 | PNMA family member 2 | Q9UL42 | HPA001936 |
| POU3F2 | Pou class 3 homeobox 2 | P20265 | HPA065187 |
| PRRT2 | Proline rich transmembrane protein 2 | Q7Z6L0 | HPA019203 |
| PRRT2 | Proline rich transmembrane protein 2 | Q7Z6L0 | HPA048045 |
| PTPN5 | Protein tyrosine phosphatase, non-receptor type 5 | P54829 | HPA031014 |
| PTPRD | Protein tyrosine phosphatase, receptor type D | P23468 | HPA054829 |
| RASL10A | RAS like family 10 member A | Q92737 | HPA056169 |
| RPH3A | Rabphilin 3A | Q9Y2J0 | HPA002475 |
| RTN1 | Reticulon 1 | Q16799 | HPA044249 |
| S100B | S100 calcium binding protein B | P04271 | HPA015768 |
| SCN2A | Sodium voltage-gated channel alpha subunit 2 | Q99250 | HPA067350 |
| SEPT_3 | Septin 3 | Q9UH03 | HPA003548 |
| SEZ6 | Seizure related 6 homolog | Q53EL9 | HPA012067 |
| SLC12A5 | Solute carrier family 12 member 5 | Q9H2X9 | HPA072058 |
| SLC17A6 | Solute carrier family 17 member 6 | Q9P2U8 | HPA039226 |
| SLC17A7 | Solute carrier family 17 member 7 | Q9P2U7 | HPA050458 |
| SLC17A7 | Solute carrier family 17 member 7 | Q9P2U7 | HPA063679 |
| SLC1A2 | Solute carrier family 1 member 2 | P43004 | HPA009172 |
| SLC32A1 | Solute carrier family 32 member 1 | Q9H598 | HPA058859 |
| SLC32A1 | Solute carrier family 32 member 1 | Q9H598 | HPA059985 |
| SLC35F1 | Solute carrier family 35 member F1 | Q5T1Q4 | HPA019576 |
| SLC39A12 | Solute carrier family 39 member 12 | Q504Y0 | HPA077034 |
| SLC39A12 | Solute carrier family 39 member 12 | Q504Y0 | HPA077354 |
| SLC4A10 | Solute carrier family 4 member 10 | Q6U841 | HPA034755 |
| SLITRK1 | SLIT and NTRK like family member 1 | Q96PX8 | HPA012414 |
| SNCB | Synuclein beta | Q16143 | HPA035876 |
| SOX11 | SRY-box 11 | P35716 | HPA000448 |
| SPP1 | Secreted phosphoprotein 1 | P10451 | HPA027541 |
| SPTAN1 | Spectrin alpha non-erythrocytic 1 | Q13813 | HPA007927 |
| SPTBN1 | Spectrin beta non-erythrocytic 1 | Q01082 | HPA013149 |
| STMN4 | Stathmin 4 | Q9H169 | HPA078407 |
| STX3 | Syntaxin 3 | Q13277 | HPA069176 |
| SV2A | Synaptic vesicle glycoprotein 2A | Q7L0J3 | HPA007863 |
| SYN1 | Synapsin 1 | P17600 | HPA000397 |
| SYT1 | Synaptotagmin 1 | P21579 | HPA064788 |
| SYT11 | Synaptotagmin 11 | Q9BT88 | HPA064091 |
| TBR1 | T-box brain 1 | Q16650 | HPA078657 |
| TGFB1 | Transforming growt factor beta 1 | P01137 | HPA047516 |
| TGFB2 | Transforming growth factor beta 2 | P61812 | HPA065065 |
| TMEM132D | Transmembrane protein 132D | Q14C87 | HPA010739 |
| TMEM151A | Transmembrane protein 151A | Q8N4L1 | HPA041035 |
| TMEM59L | Transmembrane protein 59 like | Q9UK28 | HPA010661 |
| TNF | Tumor necrosis factor | P01375 | HPA050631 |
| TNF | Tumor necrosis factor | P01375 | HPA077901 |
| TNNI2 | Troponin I2, fast skeletal type | P48788 | HPA055938 |
| TNNT2 | Troponin T2 cardiac type | P45379 | HPA017888 |
| TPH1 | Tryptophan hydroxylase 1 | P17752 | HPA022483 |
| TRIM9 | Tripartite motif contining 9 | Q9C026 | HPA067525 |
| TTC9B | Tetratricopeptide repeat domain 9B | Q8N6N2 | HPA042496 |
| TUBB1 | Tubulin beta 1 class VI | Q9H4B7 | HPA043640 |
| VCAM1 | Vascular cell adhesion molecule 1 | P19320 | HPA069867 |
| VEGFA | Vascular endothelial growth factor A | P15692 | HPA069116 |
| VEGFB | Vascular endothelial growth factor B | P49765 | HPA059415 |
| VEGFC | Vascular endothelial growth factor C | P49767 | HPA004138 |
| VEGFC | Vascular endothelial growth factor C | P49767 | HPA073518 |
| VSTM2B | V-set and transmembrane domain containing 2B | A6NLU5 | HPA073612 |
| VWC2L | Von Willebrand factor C domain containing protein 2 like | B2RUY7 | HPA044815 |
| ZDHHC22 | Zinc finger DHHC-type containing 22 | Q8N966 | HPA062500 |
| Protein and antibodies included in the study. Proteins are abbreviated across the manuscript utilizing their abbreviated gene name. All proteins had a corresponding antibody, of importance as different antibodies on the same protein do not necessarily yield similar results. Antibody ID correspond to the antibody names used in the Human Protein Atlas, where also the full protein name was derived from. | | | |

| **Supplementary Table 2 (S2): Low antibody correlations between compartments** | |
| --- | --- |
| **Protein, Antibody** | **Correlation coefficient Spearman ρ** |
| OLIG1 HPA077730 | -0.314 |
| IL6 HPA064428 | -0.225 |
| CXCL1 HPA067614 | -0.157 |
| GFAP HPA056030 | -0.129 |
| SLC32A1 HPA059985 | -0.106 |
| AK5 HPA019128 | -0.101 |
| CFB HPA001817 | -0.08 |
| CNTNAP4 HPA057342 | -0.078 |
| NEFL HPA014850 | -0.063 |
| ZDHHC22 HPA062500 | -0.057 |
| ENO2 HPA078378 | -0.055 |
| ACVR1 HPA008014 | -0.053 |
| FCN1 HPA001295 | -0.051 |
| AQP4 HPA014784 | -0.041 |
| NRXN1 HPA059963 | -0.034 |
| NEUROD2 HPA049077 | -0.027 |
| ENO2 HPA070138 | -0.024 |
| APP HPA001462 | -0.02 |
| HTR2A HPA014011 | -0.018 |
| PACSIN1 HPA028852 | -0.01 |
| MAPT HPA069570 | -0.008 |
| IL4 HPA042270 | -0.007 |
| Analytes (protein/antibody) for which between-compartment (CSF/serum) correlation ρ < 0. **Abbreviations:** CSF, cerebrospinal fluid. Full protein names are detailed in **Table S1**. | |

| **Supplementary Table 3 (S3): Protein level alterations following TBI** | | | | | | |
| --- | --- | --- | --- | --- | --- | --- |
| **Protein Antibody** | **Compartment** | **Altered CSF & serum** | **Highest Tissue Enrichment** | **Protein Function** | **𝚫MFI** | **Adj p-value** |
| DSCAM HPA074915 | CSF | 1 | cns | neurological | 5001.8 | < 0.001 |
| MBP HPA049222 | CSF | 1 | cns | neurological | 3655.2 | < 0.001 |
| CASKIN1 HPA076882 | CSF | 1 | cns |  | 2278.9 | 0.0018 |
| AQP4 HPA014944 | CSF | 0 | cns | neurological | 2207.6 | 0.0023 |
| CFB HPA001817 | Serum | 0 | liver/gallbladder | immunesystem | 2130.6 | < 0.001 |
| C9 HPA029577 | Serum | 0 | liver/gallbladder | immunesystem | 1999.9 | < 0.001 |
| PCDHGC5 HPA076140 | CSF | 0 | cns | neurological | 1546.6 | 0.0052 |
| GFAP HPA063513 | CSF | 0 | cns | neurological | 1470.7 | < 0.001 |
| OLIG1 HPA077217 | CSF | 0 | cns | neurological | 1139.8 | 0.0065 |
| C9 HPA070709 | Serum | 0 | liver/gallbladder | immunesystem | 1126.2 | < 0.001 |
| SLC39A12 HPA077034 | CSF | 0 | cns | homeostasis/signaling | 955.4 | 0.0017 |
| CASKIN1 HPA076882 | Serum | 1 | cns |  | 927.7 | < 0.001 |
| DSCAM HPA074915 | Serum | 1 | cns | neurological | 824.4 | < 0.001 |
| MASP2 HPA029313 | CSF | 1 | liver/gallbladder | immunesystem | 769.5 | 0.0044 |
| SNCB HPA035876 | CSF | 1 | cns | neurological | 626.3 | 0.0145 |
| MASP2 HPA029314 | Serum | 1 | liver/gallbladder | immunesystem | 568.2 | < 0.001 |
| MMP9 HPA063909 | Serum | 1 | bonemarrow/lymphoid | homeostasis/breakdown | 429.9 | < 0.001 |
| C5 HPA075945 | CSF | 0 | liver/gallbladder | immunesystem | 423.1 | 0.0065 |
| S100B HPA015768 | CSF | 0 | cns | homeostasis/intracellular | 395.8 | < 0.001 |
| CFI HPA001143 | CSF | 1 | liver/gallbladder | immunesystem | 373.5 | < 0.001 |
| SLC35F1 HPA019576 | CSF | 1 | cns | homeostasis/signaling | 324 | < 0.001 |
| BTBD17 HPA025022 | CSF | 0 | cns |  | 312.5 | < 0.001 |
| MMP9 HPA001238 | Serum | 1 | bonemarrow/lymphoid | homeostasis/breakdown | 311 | < 0.001 |
| RTN1 HPA044249 | CSF | 0 | cns | neurological | 301.1 | < 0.001 |
| ARPP21 HPA017303 | CSF | 0 | cns | neurological | 299 | 0.0073 |
| IL6 HPA064428 | Serum | 0 | adipose/soft tissue | immunesystem | 278.1 | 0.004 |
| SPTAN1 HPA007927 | CSF | 0 | cns | homeostasis/intracellular | 268.8 | < 0.001 |
| MASP1 HPA001617 | CSF | 0 | liver/gallbladder | immunesystem | 255.1 | 0.0205 |
| TNNI2 HPA055938 | Serum | 1 | muscle | muscle | 242.7 | < 0.001 |
| GFAP HPA056030 | CSF | 0 | cns | neurological | 241 | < 0.001 |
| FRMPD4 HPA075921 | CSF | 0 | endocrine | neurological | 239.8 | 0.0035 |
| PCDHGB1 HPA076182 | CSF | 0 | cns | neurological | 220.4 | 0.0118 |
| FCN3 HPA071173 | CSF | 0 | lung | immunesystem | 209.3 | 0.0023 |
| MASP2 HPA029313 | Serum | 1 | liver/gallbladder | immunesystem | 199.5 | < 0.001 |
| IL1A HPA075911 | CSF | 0 | bonemarrow/lymphoid | immunesystem | 197.7 | 0.0204 |
| FCN1 HPA001295 | Serum | 1 | blood | immunesystem | 191.5 | 0.0207 |
| ACVR1 HPA008014 | CSF | 0 | female genital | homeostasis/intracellular | 189.3 | < 0.001 |
| NTSR2 HPA007320 | CSF | 0 | cns | neurological | 181.4 | 0.0344 |
| ERMN HPA038295 | CSF | 0 | cns | neurological | 174.8 | < 0.001 |
| MASP2 HPA029314 | CSF | 1 | liver/gallbladder | immunesystem | 172.8 | 0.0035 |
| FCN1 HPA000685 | Serum | 1 | blood | immunesystem | 170 | 0.0437 |
| MAPT HPA069524 | CSF | 0 | cns | neurological | 167.6 | < 0.001 |
| STX3 HPA069176 | CSF | 0 | eye | neurological | 161.2 | 0.0086 |
| CFI HPA001143 | Serum | 1 | liver/gallbladder | immunesystem | 153.4 | 0.0024 |
| NKAIN2 HPA035136 | CSF | 0 | cns | homeostasis/intracellular | 151.5 | 0.0483 |
| HAPLN2 HPA045765 | CSF | 0 | cns | neurological | 149.6 | 0.0075 |
| AK5 HPA019128 | CSF | 1 | cns | neurological | 138.8 | 0.0063 |
| GRM1 HPA015701 | CSF | 1 | cns | neurological | 138.1 | < 0.001 |
| ALDOC HPA003282 | Serum | 0 | cns | neurological | 133.2 | 0.0018 |
| GRIN1 HPA067773 | CSF | 0 | cns | neurological | 131.5 | 0.0037 |
| FCN1 HPA000685 | CSF | 1 | blood | immunesystem | 131.2 | < 0.001 |
| GRIN2A HPA004693 | CSF | 0 | cns | neurological | 130.3 | < 0.001 |
| SEPT_3 HPA003548 | CSF | 0 | cns | homeostasis | 128.8 | < 0.001 |
| TUBB1 HPA043640 | CSF | 0 | blood | homeostasis/intracellular | 124.7 | < 0.001 |
| GRM1 HPA015701 | Serum | 1 | cns | neurological | 123.7 | 0.002 |
| MMP9 HPA063909 | CSF | 1 | bonemarrow/lymphoid | homeostasis/breakdown | 122.3 | 0.0048 |
| AVP HPA071892 | CSF | 0 | cns | neurological | 121.1 | < 0.001 |
| TGFB2 HPA065065 | CSF | 0 | male genital | homeostasis/signaling | 119.9 | 0.0406 |
| TRIM9 HPA067525 | CSF | 0 | cns | homeostasis/breakdown | 119.6 | < 0.001 |
| KLK6 HPA019525 | CSF | 0 | cns | homeostasis/breakdown | 115.6 | 0.0053 |
| KIF3C HPA075785 | CSF | 0 | cns | homeostasis/signaling | 114.9 | < 0.001 |
| AMER2 HPA039458 | CSF | 0 | cns | neurological | 112.2 | < 0.001 |
| SLC35F1 HPA019576 | Serum | 1 | cns | homeostasis/signaling | 110.3 | 0.0021 |
| MBP HPA049222 | Serum | 1 | cns | neurological | 110 | 0.005 |
| C1orf61 HPA076077 | CSF | 0 | cns | neurological | 107.3 | < 0.001 |
| TGFB1 HPA047516 | CSF | 0 | bonemarrow/lymphoid | homeostasis/signaling | 96.8 | 0.0043 |
| ENO2 HPA078378 | Serum | 0 | cns | neurological | 96.3 | 0.024 |
| MMP9 HPA001238 | CSF | 1 | bonemarrow/lymphoid | homeostasis/breakdown | 87.3 | < 0.001 |
| SPTBN1 HPA013149 | CSF | 0 | endocrine | homeostasis/intracellular | 86.3 | < 0.001 |
| GPM6B HPA077843 | CSF | 0 | cns | neurological | 76.8 | 0.0053 |
| FAM181B HPA075523 | CSF | 0 | cns |  | 74.9 | 0.0044 |
| SLC39A12 HPA077354 | CSF | 0 | cns | homeostasis/signaling | 72.8 | 0.0316 |
| STMN4 HPA078407 | CSF | 0 | cns |  | 68.3 | 0.0172 |
| HIF3A HPA041141 | CSF | 0 | female genital | homeostasis/hypoxia | 67.5 | < 0.001 |
| HTR2C HPA052903 | CSF | 0 | cns | neurological | 63.8 | < 0.001 |
| DSCAM HPA019324 | CSF | 0 | cns | neurological | 63.5 | 0.0092 |
| ACVR1C HPA007982 | CSF | 0 | adipose/soft tissue | homeostasis/apoptosis | 58.8 | 0.0077 |
| KCNV1 HPA069362 | CSF | 0 | cns | homeostasis/signaling | 55.6 | 0.0062 |
| FCN1 HPA001295 | CSF | 1 | blood | immunesystem | 52.3 | 0.0042 |
| POU3F2 HPA065187 | Serum | 1 | cns | neurological | 52.3 | 0.0051 |
| PNMA2 HPA001936 | CSF | 0 | cns |  | 51.6 | < 0.001 |
| CHRNB2 HPA062865 | CSF | 0 | cns | neurological | 50.6 | 0.0011 |
| HPCA HPA043245 | CSF | 0 | cns | neurological | 49.7 | 0.0451 |
| SOX11 HPA000448 | CSF | 0 | cns | neurological | 49.2 | 0.0219 |
| OLIG2 HPA003254 | CSF | 0 | cns | neurological | 48.7 | 0.0033 |
| IL1B HPA064606 | CSF | 0 | bonemarrow/lymphoid | immunesystem | 48.5 | < 0.001 |
| APC2 HPA078002 | CSF | 0 | cns | homeostasis/intracellular | 48.4 | 0.0088 |
| BAALC HPA077738 | CSF | 0 | cns | neurological | 47.8 | < 0.001 |
| HSPA4 HPA010023 | CSF | 0 | female genital |  | 47.4 | 0.0053 |
| NEFL HPA014850 | CSF | 0 | cns | neurological | 46 | < 0.001 |
| TNNI2 HPA055938 | CSF | 1 | muscle | muscle | 42.7 | < 0.001 |
| LRTM2 HPA062745 | CSF | 0 | cns |  | 38.8 | 0.0243 |
| NR2E1 HPA055642 | CSF | 0 | cns | neurological | 38.8 | 0.0207 |
| CAMK2G HPA051785 | CSF | 0 | muscle | muscle | 36.2 | 0.0034 |
| LRRTM4 HPA061911 | CSF | 0 | cns | neurological | 35.7 | 0.0105 |
| CACNG8 HPA041351 | CSF | 0 | cns | homeostasis/signaling | 35.5 | 0.0126 |
| KCNJ9 HPA070478 | CSF | 0 | cns | homeostasis/signaling | 35.3 | 0.0224 |
| NEFH HPA061615 | CSF | 1 | male genital | neurological | 31.7 | 0.0018 |
| C2orf80 HPA078078 | CSF | 0 | cns |  | 31.5 | 0.019 |
| PTPN5 HPA031014 | CSF | 0 | cns | neurological | 31 | 0.0186 |
| CSPG5 HPA076601 | CSF | 0 | cns | neurological | 29.3 | < 0.001 |
| POU3F2 HPA065187 | CSF | 1 | cns | neurological | 29.3 | 0.0459 |
| KCNF1 HPA014738 | CSF | 0 | cns | homeostasis/signaling | 29.1 | 0.0171 |
| CXCL1 HPA067614 | CSF | 1 | bonemarrow/lymphoid | immunesystem | 29 | 0.0246 |
| GPM6A HPA017338 | CSF | 0 | cns | neurological | 28.7 | 0.0494 |
| DLL3 HPA060025 | CSF | 0 | cns | neurological | 28.3 | 0.0474 |
| JPH3 HPA076304 | CSF | 0 | cns | neurological | 27.4 | 0.0346 |
| LHFPL3 HPA077221 | CSF | 0 | cns |  | 26.2 | < 0.001 |
| PACSIN1 HPA028852 | CSF | 0 | cns | neurological | 25.2 | < 0.001 |
| GRM3 HPA053434 | CSF | 0 | cns | neurological | 23.2 | 0.0211 |
| MAPT HPA069570 | CSF | 0 | cns | neurological | 22.8 | 0.0459 |
| IL4 HPA070010 | CSF | 0 | blood | immunesystem | 21.6 | 0.027 |
| HIF1A HPA001275 | CSF | 0 | bonemarrow/lymphoid | homeostasis/hypoxia | 21.4 | 0.012 |
| ELAVL3 HPA070436 | CSF | 1 | cns | neurological | 20.2 | 0.0491 |
| CNTNAP4 HPA057342 | Serum | 1 | cns | neurological | 19.8 | 0.0337 |
| TMEM151A HPA041035 | CSF | 0 | cns |  | 17.2 | 0.0194 |
| SYT1 HPA064788 | CSF | 0 | cns | neurological | 14.8 | 0.0287 |
| FCN2 HPA076099 | CSF | 0 | liver/gallbladder | immunesystem | 13.2 | 0.0065 |
| SNCB HPA035876 | Serum | 1 | cns | neurological | -26.1 | 0.0318 |
| ALDOC HPA067442 | Serum | 0 | cns | neurological | -27.2 | 0.0435 |
| OLFM1 HPA057444 | Serum | 1 | cns | neurological | -40.3 | 0.0243 |
| VEGFC HPA073518 | CSF | 0 | female genital | development | -43.3 | 0.0326 |
| NEFH HPA061615 | Serum | 1 | male genital | neurological | -46.2 | 0.0144 |
| SLC1A2 HPA009172 | CSF | 0 | cns | neurological | -55.8 | < 0.001 |
| SYN1 HPA000397 | Serum | 0 | cns | neurological | -64.6 | 0.0118 |
| PRRT2 HPA019203 | CSF | 0 | cns | neurological | -86.7 | 0.0232 |
| CXCL1 HPA067614 | Serum | 1 | bonemarrow/lymphoid | immunesystem | -95.9 | 0.0035 |
| GALNT17 HPA013624 | CSF | 0 | cns | homeostasis/intracellular | -103.6 | < 0.001 |
| RPH3A HPA002475 | CSF | 0 | cns | neurological | -104.9 | 0.0324 |
| VEGFC HPA004138 | Serum | 0 | female genital | development | -112.3 | 0.0132 |
| KCNC1 HPA041392 | Serum | 0 | cns | neurological | -115.9 | 0.0148 |
| ZDHHC22 HPA062500 | Serum | 0 | cns |  | -138.9 | 0.0459 |
| GDAP1L1 HPA063265 | Serum | 0 | cns | neurological | -140.1 | 0.0017 |
| OLFM1 HPA057444 | CSF | 1 | cns | neurological | -154.7 | < 0.001 |
| NEUROD2 HPA049077 | Serum | 0 | cns | neurological | -157.7 | 0.0244 |
| SLITRK1 HPA012414 | CSF | 0 | cns | neurological | -163.3 | < 0.001 |
| VEGFA HPA069116 | Serum | 0 | endocrine | development | -165.4 | < 0.001 |
| SYT11 HPA064091 | CSF | 0 | cns | neurological | -170.9 | 0.0027 |
| RASL10A HPA056169 | Serum | 0 | cns | homeostasis/intracellular | -185.1 | 0.0175 |
| ELAVL3 HPA070436 | Serum | 1 | cns | neurological | -187.9 | 0.0171 |
| GPR37L1 HPA064454 | Serum | 1 | cns | homeostasis/signaling | -217.8 | < 0.001 |
| GPR26 HPA062736 | Serum | 0 | cns | homeostasis/signaling | -229.7 | 0.0224 |
| AK5 HPA019128 | Serum | 1 | cns | neurological | -238 | 0.0129 |
| PRRT2 HPA048045 | CSF | 0 | cns | neurological | -286.6 | 0.0027 |
| SLC12A5 HPA072058 | CSF | 0 | cns | neurological | -367.1 | 0.027 |
| TMEM132D HPA010739 | CSF | 0 | cns | neurological | -402.3 | < 0.001 |
| HRH3 HPA072396 | Serum | 0 | cns | homeostasis/signaling | -429.9 | < 0.001 |
| CNTNAP4 HPA057342 | CSF | 1 | cns | neurological | -447.2 | < 0.001 |
| CSPG5 HPA071779 | CSF | 0 | cns | neurological | -543.9 | < 0.001 |
| MOG HPA021873 | CSF | 0 | cns | neurological | -593.2 | 0.0018 |
| BCAN HPA007865 | CSF | 0 | cns | neurological | -679.1 | < 0.001 |
| VSTM2B HPA073612 | CSF | 0 | cns |  | -704.7 | < 0.001 |
| PDYN HPA053342 | CSF | 0 | cns | neurological | -815.7 | < 0.001 |
| NCAN HPA077060 | CSF | 0 | cns | neurological | -987.5 | < 0.001 |
| GPR37L1 HPA064454 | CSF | 1 | cns | homeostasis/signaling | -1129.5 | < 0.001 |
| APP HPA001462 | CSF | 0 | cns | neurological | -2398 | < 0.001 |
| NPTX1 HPA077062 | CSF | 0 | cns | neurological | -4575.8 | < 0.001 |
| MEPE HPA071946 | Serum | 0 | cns | bone | -4764.4 | < 0.001 |
| Following TBI, numerous proteins exhibited significantly different MFI values compared with control subjects. Protein level differences were assessed using the Wilcoxon-Rank Sum Test. Δ MFI was calculated as median MFI_TBI_ – median MFI_control_, meaning that Δ MFI > 0 equals increased protein levels following TBI and conversely, Δ MFI < 0 denotes decreased protein levels following TBI. **Abbreviations:** CSF, cerebrospinal fluid; CNS, central nervous system; MFI, median fluorescence intensity. Full protein names are detailed in **Table S1**. | | | | | | |

| **Supplementary Table 4 (S4): Proteins with CSF/serum ratio significantly correlated with Q_A_** | | | | | |
| --- | --- | --- | --- | --- | --- |
| **Protein, Antibody** | **Highest tissue enrichment** | **Function group** | **Function specific** | **Kendall 𝛕** | **adjusted p-value** |
| C1QB HPA052116 | bonemarrow/lymphoid | immunesystem | innate_immunity | 0.67 | <0.001 |
| CFB HPA001817 | liver/gallbladder | immunesystem | innate_immunity | 0.66 | <0.001 |
| C9 HPA029577 | liver/gallbladder | immunesystem | complement_system | 0.65 | <0.001 |
| C9 HPA070709 | liver/gallbladder | immunesystem | complement_system | 0.65 | <0.001 |
| C1QA HPA002350 | bonemarrow/lymphoid | immunesystem | innate_immunity | 0.64 | <0.001 |
| MASP2 HPA029314 | liver/gallbladder | immunesystem | complement_system | 0.58 | <0.001 |
| VCAM1 HPA069867 | bonemarrow/lymphoid | immunesystem | cell_cell_communication | 0.54 | <0.001 |
| FCN3 HPA071173 | lung | immunesystem | innate_immunity | 0.54 | <0.001 |
| MASP2 HPA029313 | liver/gallbladder | immunesystem | complement_system | 0.52 | <0.001 |
| C5 HPA075945 | liver/gallbladder | immunesystem | innate_immunity | 0.52 | <0.001 |
| DSCAM HPA074915 | cns | neurological | neural_development | 0.51 | <0.001 |
| ENO2 HPA070138 | cns | neurological | neural_homeostasis | 0.49 | <0.001 |
| SLC39A12 HPA077034 | cns | homeostasis_signaling | physiology | 0.48 | <0.001 |
| CFI HPA001143 | liver/gallbladder | immunesystem | complement_system | 0.46 | <0.001 |
| ACVR1 HPA007505 | female genital | homeostasis_intracellular | intracellular_signaling | 0.46 | <0.001 |
| ENO2 HPA078378 | cns | neurological | neural_homeostasis | 0.46 | <0.001 |
| MEPE HPA071946 | cns | bone | ecm | 0.45 | <0.001 |
| SLC35F1 HPA019576 | cns | homeostasis_signaling | solute_transporter | 0.44 | <0.001 |
| GDAP1L1 HPA063265 | cns | neurological | neural_development | 0.44 | <0.001 |
| IL1B HPA068737 | bonemarrow/lymphoid | immunesystem | proinflammatory | 0.44 | <0.001 |
| GAP43 HPA013392 | cns | neurological | neural_development | 0.43 | <0.001 |
| TGFB1 HPA047516 | bonemarrow/lymphoid | homeostasis_signaling | intracellular_signaling | 0.43 | <0.001 |
| PRRT2 HPA048045 | cns | neurological | neurotransmission | 0.43 | <0.001 |
| IL6 HPA064428 | adipose/soft tissue | immunesystem | acute_phase_response | 0.43 | <0.001 |
| CASKIN1 HPA076882 | cns |  |  | 0.43 | <0.001 |
| APP HPA031303 | cns | neurological | cell_surface_receptor | 0.42 | <0.001 |
| VEGFC HPA073518 | female genital | development | angiogenesis | 0.42 | <0.001 |
| MCHR2 HPA050708 | cns | endocrine | melatonin | 0.41 | <0.001 |
| SLC12A5 HPA072058 | cns | neurological | neurotransmission | 0.41 | <0.001 |
| FCN1 HPA001295 | blood | immunesystem | innate_immunity | 0.4 | <0.001 |
| ERMN HPA038296 | cns | neurological | neural_homeostasis | 0.4 | <0.001 |
| GRIN1 HPA067773 | cns | neurological | neural_physiology | 0.4 | <0.001 |
| STX3 HPA069176 | eye | neurological | neurotransmission | 0.4 | <0.001 |
| APP HPA001462 | cns | neurological | cell_surface_receptor | 0.39 | <0.001 |
| FCN1 HPA000685 | blood | immunesystem | innate_immunity | 0.39 | <0.001 |
| ALDOC HPA003282 | cns | neurological | neural_metabolism | 0.39 | <0.001 |
| BCAN HPA007865 | cns | neurological | neural_development | 0.39 | <0.001 |
| NETO1 HPA073068 | cns | neurological | neural_development | 0.39 | <0.001 |
| AQP4 HPA014784 | cns | neurological | neural_homeostasis | 0.38 | <0.001 |
| SNCB HPA035876 | cns | neurological | neural_homeostasis | 0.38 | <0.001 |
| NPTX1 HPA077062 | cns | neurological | neurotransmission | 0.38 | <0.001 |
| NCAN HPA077060 | cns | neurological | neural_development | 0.38 | <0.001 |
| NEFL HPA014850 | cns | neurological | neural_homeostasis | 0.37 | <0.001 |
| BTBD17 HPA025022 | cns |  |  | 0.37 | <0.001 |
| ERC2 HPA073739 | cns | neurological | neurotrasmitter_release | 0.37 | <0.001 |
| RTN1 HPA044249 | cns | neurological | neuroendocrine_signaling | 0.36 | <0.001 |
| ALDOC HPA067442 | cns | neurological | neural_metabolism | 0.36 | <0.001 |
| MAPT HPA069524 | cns | neurological | cytoskeleton | 0.36 | <0.001 |
| NTSR2 HPA077042 | cns | neurological | intracellular_signaling | 0.36 | <0.001 |
| SEPT_3 HPA003548 | cns | homeostasis |  | 0.35 | <0.001 |
| HTR2A HPA014011 | cns | neurological | neurotransmission | 0.35 | <0.001 |
| S100B HPA015768 | cns | homeostasis_intracellular | intracellular_signaling | 0.35 | <0.001 |
| NRXN1 HPA059963 | cns | neurological | cell_cell_communication | 0.35 | <0.001 |
| MMP9 HPA063909 | bonemarrow/lymphoid | homeostasis_breakdown | ecm | 0.35 | <0.001 |
| FEZF2 HPA068604 | cns | neurological | neural_development | 0.35 | <0.001 |
| SPTAN1 HPA007927 | cns | homeostasis_intracellular | intracellular_signaling | 0.34 | <0.001 |
| KCNC1 HPA041392 | cns | neurological | ion_channel | 0.34 | <0.001 |
| CNTNAP4 HPA057342 | cns | neurological | neurotransmission | 0.34 | <0.001 |
| MBP HPA073581 | cns | neurological | myelin_component | 0.34 | <0.001 |
| STMN4 HPA078407 | cns |  |  | 0.34 | <0.001 |
| VEGFC HPA004138 | female genital | development | angiogenesis | 0.33 | <0.001 |
| MASP1 HPA001617 | liver/gallbladder | immunesystem | innate_immunity | 0.33 | 0.001 |
| ACVR1 HPA008014 | female genital | homeostasis_intracellular | intracellular_signaling | 0.33 | 0.001 |
| SLITRK1 HPA012414 | cns | neurological | neural_development | 0.33 | <0.001 |
| ARPP21 HPA017303 | cns | neurological | neurotransmission | 0.33 | 0.001 |
| FABP7 HPA061703 | cns | neurological | neural_development | 0.33 | <0.001 |
| IL1B HPA064606 | bonemarrow/lymphoid | immunesystem | proinflammatory | 0.33 | 0.001 |
| TRIM9 HPA067525 | cns | homeostasis_breakdown | degradation | 0.33 | 0.001 |
| TBR1 HPA078657 | cns | neurological | neural_development | 0.33 | <0.001 |
| RPH3A HPA002475 | cns | neurological | neurotrasmitter_release | 0.32 | 0.003 |
| GRIN2A HPA004693 | cns | neurological | ion_channel | 0.32 | 0.002 |
| SLC1A2 HPA009172 | cns | neurological | neurotransmission | 0.32 | 0.002 |
| OPALIN HPA014372 | cns |  |  | 0.32 | 0.002 |
| BAALC HPA027132 | cns | neurological | neurotransmission | 0.32 | 0.002 |
| ERMN HPA038295 | cns | neurological | neural_homeostasis | 0.32 | 0.002 |
| HPCA HPA043245 | cns | neurological | ion_channel | 0.32 | 0.002 |
| DIRAS2 HPA043758 | cns |  |  | 0.32 | 0.002 |
| HAPLN2 HPA045765 | cns | neurological | neural_development | 0.32 | 0.002 |
| C8orf46 HPA075134 | cns | neurological | neural_development | 0.32 | 0.003 |
| SYN1 HPA000397 | cns | neurological | neurotrasmitter_release | 0.31 | 0.004 |
| GRM1 HPA015701 | cns | neurological | neurotransmission | 0.31 | 0.004 |
| GABRG1 HPA035622 | cns | neurological | neurotransmission | 0.31 | 0.003 |
| GFAP HPA056030 | cns | neurological | cytoskeleton | 0.31 | 0.003 |
| OLFM1 HPA057444 | cns | neurological | neural_development | 0.31 | 0.004 |
| OPCML HPA065374 | cns | homeostasis_signaling | signaling | 0.31 | 0.004 |
| FAM181B HPA066861 | cns |  |  | 0.31 | 0.003 |
| VEGFA HPA069116 | endocrine | development | angiogenesis | 0.31 | 0.004 |
| NTSR2 HPA007320 | cns | neurological | intracellular_signaling | 0.3 | 0.005 |
| SPTBN1 HPA013149 | endocrine | homeostasis_intracellular | intracellular_signaling | 0.3 | 0.006 |
| SPP1 HPA027541 | urinary tract | homeostasis_signaling | cell_cell_communication | 0.3 | 0.007 |
| GFAP HPA063513 | cns | neurological | cytoskeleton | 0.3 | 0.006 |
| TMEM132D HPA010739 | cns | neurological | neural_development | 0.29 | 0.011 |
| AQP4 HPA014944 | cns | neurological | neural_homeostasis | 0.29 | 0.009 |
| PRRT2 HPA019203 | cns | neurological | neurotransmission | 0.29 | 0.008 |
| MOG HPA021873 | cns | neurological | cell_cell_communication | 0.29 | 0.01 |
| C11orf87 HPA034656 | cns |  |  | 0.29 | 0.008 |
| TTC9B HPA042496 | cns |  |  | 0.29 | 0.012 |
| PDYN HPA049841 | cns | neurological | neural_physiology | 0.29 | 0.012 |
| TGFB2 HPA065065 | male genital | homeostasis_signaling | signaling | 0.29 | 0.01 |
| CSPG5 HPA071779 | cns | neurological | neural_development | 0.29 | 0.012 |
| AVP HPA071892 | cns | neurological | neuroendocrine_signaling | 0.29 | 0.009 |
| HRH3 HPA072396 | cns | homeostasis_signaling | signaling | 0.29 | 0.008 |
| SV2A HPA007863 | cns | neurological | neurotransmission | 0.28 | 0.019 |
| DSCAM HPA019324 | cns | neurological | neural_development | 0.28 | 0.013 |
| NKAIN2 HPA035136 | cns | homeostasis_intracellular |  | 0.28 | 0.016 |
| AMER2 HPA039458 | cns | neurological | neural_development | 0.28 | 0.018 |
| CACNG3 HPA059010 | cns | neurological | intracellular_signaling | 0.28 | 0.014 |
| ZDHHC22 HPA062500 | cns |  |  | 0.28 | 0.018 |
| IL10 HPA071391 | bonemarrow/lymphoid | immunesystem | antiinflammatory | 0.28 | 0.014 |
| IL1A HPA075911 | bonemarrow/lymphoid | immunesystem | inflammation | 0.28 | 0.014 |
| SLC39A12 HPA077354 | cns | homeostasis_signaling | physiology | 0.28 | 0.018 |
| HSPA4 HPA010023 | female genital |  |  | 0.27 | 0.02 |
| TMEM59L HPA010661 | cns | neurological | intracellular_signaling | 0.27 | 0.026 |
| SEZ6 HPA012067 | cns | neurological | intracellular_signaling | 0.27 | 0.027 |
| KLK6 HPA019525 | cns | homeostasis_breakdown | intracellular_signaling | 0.27 | 0.02 |
| NEUROD2 HPA049077 | cns | neurological | neural_development | 0.27 | 0.022 |
| CLEC7A HPA050229 | blood | immunesystem | innate_immunity | 0.27 | 0.022 |
| AK5 HPA057255 | cns | neurological | intracellular_signaling | 0.27 | 0.029 |
| C1QL2 HPA057934 | cns | neurological | neurotransmission | 0.27 | 0.029 |
| SCN2A HPA067350 | cns | homeostasis_signaling | signaling | 0.27 | 0.024 |
| JPH3 HPA076304 | cns | neurological | intracellular_signaling | 0.27 | 0.026 |
| C1orf61 HPA076077 | cns | neurological | neural_homeostasis | 0.27 | 0.029 |
| TUBB1 HPA043640 | blood | homeostasis_intracellular | microtubule | 0.26 | 0.044 |
| CCL18 HPA047485 | lung | immunesystem | humoral_cell_mediated_immunity | 0.26 | 0.035 |
| PDYN HPA053342 | cns | neurological | neural_physiology | 0.26 | 0.038 |
| CASKIN1 HPA055990 | cns |  |  | 0.26 | 0.038 |
| NEFH HPA061615 | male genital | neurological | cytoskeleton | 0.26 | 0.046 |
| SYT11 HPA064091 | cns | neurological | neurotransmission | 0.26 | 0.036 |
| KCNV1 HPA075000 | cns | homeostasis_signaling | ion_channel | 0.26 | 0.043 |
| OLIG2 HPA003254 | cns | neurological | neural_development | 0.25 | 0.048 |
| CACNG8 HPA041351 | cns | homeostasis_signaling | ion_channel | 0.25 | 0.048 |
| ATP6V1G2 HPA068667 | cns | homeostasis_intracellular | intracellular_signaling | 0.25 | 0.048 |
| For each protein CSF/serum ratio, a correlation analyses against Q_A_ was conducted using Kendall correlation. This applied only to TBI patients, as Q_A_ was missing for all control subjects. Proteins significantly correlated with Q_A_ are highlighted above. Notably, the proteins with highest Kendall τ were all complement proteins. **Abbreviations:** CSF, cerebrospinal fluid; CNS, central nervous system; ECM, extracellular matrix; Enrich., enrichment, Q_A_, albumin quotient. Full protein names are detailed in **Table S1**. | | | | | |

| **Supplementary Table 5 (S5): Cluster-derived proteins in CSF together with CSF and serum proteins altered following TBI comprise (novel) independent outcome predictors.** | | | | | | |
| --- | --- | --- | --- | --- | --- | --- |
| **Protein Antibody** | **Compartment** | **Highest tissue enrichment** | **Funtion** | **Regression Coefficient** | **𝚫R^2^** | **adjusted p-value** |
| STMN4 HPA078407 | CSF | cns |  | -0.00505 | 0.121 | 0.04548 |
| C5 HPA075945 | CSF | liver/gallbladder | immunesystem | -0.00095 | 0.106 | 0.04548 |
| GPR26 HPA062736 | CSF | cns | homeostasis_signaling | -0.00684 | 0.099 | 0.04548 |
| ARPP21 HPA017303 | CSF | cns | neurological | -0.00084 | 0.096 | 0.04548 |
| CFB HPA001817 | Serum | liver/gallbladder | immunesystem | 0.00098 | 0.092 | 0.04548 |
| FCN1 HPA000685 | CSF | blood | immunesystem | -0.002 | 0.092 | 0.04548 |
| C2orf80 HPA078078 | CSF | cns |  | -0.01263 | 0.088 | 0.04548 |
| CHRNB2 HPA062865 | CSF | cns | neurological | -0.00726 | 0.088 | 0.04548 |
| VEGFC HPA073518 | CSF | female genital | development | -0.008 | 0.087 | 0.04548 |
| CACNG8 HPA041351 | CSF | cns | homeostasis_signaling | -0.01099 | 0.085 | 0.04548 |
| SPTBN1 HPA013149 | CSF | endocrine | homeostasis_intracellular | -0.00091 | 0.082 | 0.04548 |
| FCN1 HPA001295 | Serum | blood | immunesystem | 0.00303 | 0.082 | 0.04548 |
| FAM181B HPA075523 | CSF | cns |  | -0.00534 | 0.08 | 0.04548 |
| HPCA HPA043245 | CSF | cns | neurological | -0.00658 | 0.08 | 0.04548 |
| SPTAN1 HPA007927 | CSF | cns | homeostasis_intracellular | -0.00045 | 0.078 | 0.04548 |
| ACVR1C HPA007982 | CSF | adipose/soft tissue | homeostasis_apoptosis | -0.00693 | 0.076 | 0.04548 |
| GRIN2A HPA004693 | CSF | cns | neurological | -0.00497 | 0.075 | 0.04548 |
| MMP9 HPA063909 | CSF | bonemarrow/lymphoid | homeostasis_breakdown | -0.00148 | 0.075 | 0.04548 |
| SEPT3 HPA003548 | CSF | cns | homeostasis | -0.00128 | 0.075 | 0.04548 |
| AMER2 HPA039458 | CSF | cns | neurological | -0.00178 | 0.075 | 0.04548 |
| C9 HPA070709 | CSF | liver/gallbladder | immunesystem | -0.00123 | 0.074 | 0.04548 |
| PTPN5 HPA031014 | CSF | cns | neurological | -0.00839 | 0.073 | 0.04548 |
| TGFB2 HPA065065 | CSF | male genital | homeostasis_signaling | -0.00248 | 0.072 | 0.04548 |
| LHFPL3 HPA077221 | CSF | cns |  | -0.01493 | 0.072 | 0.04548 |
| TGFB1 HPA047516 | CSF | bonemarrow/lymphoid | homeostasis_signaling | -0.00465 | 0.072 | 0.04548 |
| IL6 HPA064428 | Serum | adipose/soft tissue | immunesystem | 0.00185 | 0.071 | 0.04548 |
| CXCL1 HPA067614 | CSF | bonemarrow/lymphoid | immunesystem | -0.00478 | 0.071 | 0.04548 |
| NKAIN2 HPA035136 | CSF | cns | homeostasis_intracellular | -0.00252 | 0.067 | 0.04548 |
| CASKIN1 HPA076882 | CSF | cns |  | -0.00022 | 0.067 | 0.04548 |
| ZDHHC22 HPA062500 | CSF | cns |  | -0.00508 | 0.065 | 0.04548 |
| DSCAM HPA019324 | CSF | cns | neurological | -0.00446 | 0.065 | 0.04548 |
| FCN3 HPA071173 | CSF | lung | immunesystem | -0.00079 | 0.064 | 0.04924 |
| IL6 HPA064428 | CSF | adipose/soft tissue | immunesystem | -0.00302 | 0.064 | 0.04548 |
| MASP2 HPA029314 | CSF | liver/gallbladder | immunesystem | -0.00152 | 0.061 | 0.04548 |
| VEGFC HPA004138 | CSF | female genital | development | -0.00572 | 0.061 | 0.04548 |
| HAPLN2 HPA045765 | CSF | cns | neurological | -0.00281 | 0.06 | 0.04548 |
| MASP1 HPA001617 | CSF | liver/gallbladder | immunesystem | -0.00146 | 0.059 | 0.04548 |
| STX3 HPA069176 | CSF | eye | neurological | -0.00126 | 0.059 | 0.04657 |
| HTR2C HPA052903 | CSF | cns | neurological | -0.00619 | 0.059 | 0.04548 |
| RTN1 HPA044249 | CSF | cns | neurological | -0.00118 | 0.058 | 0.04895 |
| HSPA4 HPA010023 | CSF | female genital |  | -0.00282 | 0.057 | 0.04935 |
| GPM6B HPA077843 | CSF | cns | neurological | -0.00475 | 0.057 | 0.0466 |
| MASP2 HPA029313 | CSF | liver/gallbladder | immunesystem | -0.00035 | 0.056 | 0.04935 |
| NTSR2 HPA007320 | CSF | cns | neurological | -0.00249 | 0.055 | 0.04657 |
| All proteins that comprised the intersect between CSF-altered proteins and CSF cluster-derived proteins or serum-altered proteins and CSF cluster derived proteins were used for outcome analysis. Outcome prediction was conducted by univariable followed by multivariable proportional odds regression analysis where GOS was used as dependent variable and the protein level as independent variable. The IMPACT variables were used as covariates. **Abbreviations:** BBB, blood-brain barrier injury; CNS, central nervous system; Coeff., Regression Coefficient; CSF, cerebrospinal fluid; GOS, Glasgow Outcome Score; IMPACT, International Mission for Prognosis and Analysis of Clinical Trials in TBI; Q_A_, Albumin Quotient; TBI, Traumatic Brain Injury. Full protein names are listed in **Table S1**. | | | | | | |

| **Supplementary Table 6 (S6): Cluster-derived proteins in CSF together with CSF and serum proteins altered following TBI discriminate between favorable and unfavorable outcome upon multiple imputation.** | | | | | |
| --- | --- | --- | --- | --- | --- |
| **Protein, Antibody** | **Compartment** | **Highest Tissue Enrichment** | **Coefficient** | **𝚫R^2^** | **imputed p-value** |
| MBP HPA049222 | CSF | cns | 0.00025 | 0.117 | 0.002 |
| CASKIN1 HPA076882 | CSF | cns | 0.00027 | 0.093 | 0.01 |
| SNCB HPA035876 | CSF | cns | 0.00121 | 0.09 | 0.009 |
| ENO2 HPA078378 | CSF | cns | 0.00053 | 0.066 | 0.023 |
| C9 HPA029577 | CSF | liver/gallbladder | 0.00028 | 0.055 | 0.034 |
| All proteins that comprised the intersect between CSF-altered proteins and CSF cluster-derived proteins or serum-altered proteins and CSF cluster derived proteins were used for outcome analysis. Outcome prediction was conducted by univariable followed by multivariable proportional odds regression analysis where dichotomized GOS (favorable/unfavorable outcome) was used as dependent variable and the protein level as independent variable. The IMPACT variables were used as covariates. For multivariable analysis, multiple imputations were used. Reported p-values are median p-values (unadjusted) following n = 200 imputations. **Abbreviations:** BBB, blood-brain barrier injury; CNS, central nervous system; Coeff., Regression Coefficient; CSF, cerebrospinal fluid; GOS, Glasgow Outcome Score; IMPACT, International Mission for Prognosis and Analysis of Clinical Trials in TBI; Q_A_, Albumin Quotient; TBI, Traumatic Brain Injury. Full protein names are listed in **Table S1**. | | | | | |

| **Supplementary Table 7 (S7): Proteins significantly altered following BBB disruption comprise independent outcome predictors after severe TBI.** | | | | | | |
| --- | --- | --- | --- | --- | --- | --- |
| **Protein Antibody** | **Compartment** | **Highest Tissue Enrichment** | **Function** | **Regression coefficient** | **𝚫R^2^** | **adjusted p-value** |
| CFB HPA001817 | Serum | liver/gallbladder | immune system | 0.00098 | 0.092 | 0.02855 |
| FCN1 HPA001295 | Serum | blood | immune system | 0.00303 | 0.082 | 0.02855 |
| C9 HPA070709 | CSF | liver/gallbladder | immune system | -0.00123 | 0.074 | 0.03061 |
| IL6 HPA064428 | Serum | adipose/soft tissue | immune system | 0.00185 | 0.071 | 0.03061 |
| FCN1 HPA001295 | CSF | blood | immune system | -0.00365 | 0.064 | 0.03846 |
| IL6 HPA064428 | CSF | adipose/soft tissue | immune system | -0.00302 | 0.064 | 0.03061 |
| C9 HPA029577 | CSF | liver/gallbladder | immune system | -0.0003 | 0.051 | 0.04047 |
| Proteins that were significantly different between disrupted and intact BBB were subjected to a sub-group outcome analysis. Outcome prediction was conducted by univariable followed by multivariable proportional odds regression analysis where GOS was used as dependent variable and the protein level as independent variable. The IMPACT variables were used as covariates. We report model prediction improvement through Nagelkerke’s pseudo-R^2^ (𝚫R^2^). Abbreviations: BBB, blood-brain barrier injury; CNS, central nervous system; Coeff., Regression Coefficient; CSF, cerebrospinal fluid; GOS, Glasgow Outcome Score; IMPACT, International Mission for Prognosis and Analysis of Clinical Trials in TBI; TBI, Traumatic Brain Injury; Q_A_, Albumin Quotient. All full protein names are listed in **Table S1**. | | | | | | |
