# Supplementary material for: Fluid proteomics of CSF and serum reveal important neuroinflammatory proteins in blood–brain barrier disruption and outcome prediction following severe traumatic brain injury: a prospective, observational study
Source: Crit Care. 2021 Mar 12;25:103. doi: 10.1186/s13054-021-03503-x (PMC7955664; doi:10.1186/s13054-021-03503-x)
Supplement: Supplementary file 4 — Additional file 4: Supplementary methods, supplementary results, supplementary figure legends. [file 13054_2021_3503_MOESM4_ESM.docx]

Supplementary Material

Fluid Proteomics of CSF and Serum Reveal Important Neuroinflammatory Proteins in Blood-Brain Barrier Disruption and Outcome Prediction following Severe Traumatic Brain Injury: a Prospective, Observational Study

Caroline Lindblad^1^*, Elisa Pin^‡2^, David Just^‡2^, Faiez Al Nimer^1,3^, Peter Nilsson^2^, Bo-Michael Bellander^1,4^, Mikael Svensson^1,4^, Fredrik Piehl^1,3^, Eric Peter Thelin^1,5^.

1. Department of Clinical Neuroscience, Karolinska Institutet, Stockholm, Sweden
2. Division of Affinity Proteomics, Department of Protein Science, SciLifeLab, KTH-Royal Institute of Technology, Stockholm, Sweden.
3. Center for Neurology, Academic Specialist Center, Stockholm Health Services, Stockholm, Sweden.
4. Department of Neurosurgery, Karolinska University Hospital, Stockholm, Sweden.
5. Department of Neurology, Karolinska University Hospital, Stockholm, Sweden.

**Running title:** Proteomics in Human Traumatic Brain Injury

‡: These authors contributed equally to this work/shared position.

*: Corresponding author

Caroline Lindblad, MD

Karolinska Universitetssjukhuset Solna J5:20

Tema Neuro, forskargrupp Svensson

SE-171 78 Stockholm

Sweden

Telephone: +46708-236282

Email: [caroline.lindblad@ki.se](mailto:caroline.lindblad@ki.se)

Twitter: @carrolindblad

Supplementary Methods

**Quality Control Clinical Data**

CSF and serum samples were taken in adjunct to clinical sampling, why sampling time points following trauma differed between patients. Moreover, patient samples acquired from TBI patients were not immediately transferred to our biobank during weekends and evenings due to logistic drawbacks. This means that samples were stored for varying durations at 4°C. Therefore, for all patients and proteins, we calculated time from trauma to serum/CSF sample, duration before arrival in biobank, and protein content depending on duration of sample storage at 4°C.

Data on the time from trauma to sample was missing for n = 2 TBI patients (matched samples) and 1 TBI patient (non-matched samples). Data on the duration of patient sample storage before arrival to biobank was missing for n = 15 TBI (matched samples) and 7 TBI (non-matched samples). As non-matched samples (i.e. when only serum samples were taken) were incorporated for this analysis, time points differed between CSF and serum. For CSF, median time from trauma to sample was 60.8 hours (36.6-109.1) and for serum, median time from trauma to sample was 53.3 hours (30.5-91.1) (**Figure S1A**). CSF and serum samples were both stored locally in median 1 days (0-1) before biobank delivery (**Figure S1B**). Theoretically, this might affect protein content, due to proteases in body fluids, although some have argued that human serum contain low amounts of proteases (1). We therefore graphically examined protein content by patient sample and by analyte, depending on days until biobank arrival. Multiple days in fridge-storage did not seem to affect total protein content per patient sample (**Figure S2A**) or per analyte (**Figure S2B**, representative analyte example) in either CSF or serum. Notably, some analytes in general exhibited very low MFI values independent on delivery-time-to-biobank, investigated in higher detail below.

**Proteomic Assay Quality Control**

Proteomic quality control comprised bead count and intensity assessments. Bead count was assessed in all samples and for all analytes. Bead count threshold was set to 30. Individual samples with bead count < 30 or analytes with median bead count < 30 were excluded. Four CSF samples exhibited bead count <30 (**Figure S3A**), why these samples and the patients’ corresponding serum sample were excluded (n = 8 samples, n = 4 patients, in total). All analytes exhibited median bead counts ≥ 30 (**Figure S3B**).

Median fluorescence intensity measurements (median MFI) across all samples were compared. In order to compare presumptuous systematic discrepancies between CSF and serum samples, we analyzed blank, as well as pooled, samples of CSF and serum. Of note, both blank CSF and pooled CSF exhibited higher median MFI compared with the serum equivalents (**Figure S4A**, inset), thus necessitating background subtraction. Background subtraction was conducted sample-wise by sample *i* as stipulated in Equation 1 (Eq.1), i.e. the adjusted intensity was defined as the sample-wise MFI following mean bare bead subtraction, where n = 3 bare beads (*j*) per sample.

$${MFI}_{i, adjusted} ={MFI}_{i} -\left( \frac{{MFI}_{j1, i}+ {MFI}_{j2, i}+{MFI}_{j3, i}}{n_{bare beads}} \right)$$

(Eq.1)

For analytes that following background subtraction had MFI_adjusted_ ≤ 0, we added “1”, in order to enable down-stream analysis. Following background subtraction, MFI_adjusted_ was re-examined, with improved similarity between blank and pool samples (**Figure S4B**), albeit a small residual difference between Blank_CSF_ and Blank_serum_. Next, we examined MFI_adjusted_ per analyte. We found that a few analytes had median MFI values very close to 0/1 (**Figure S4C**). Analytes that exhibited median MFI_adjusted_ ≤ median MFI_rIgG_ were investigated further (n = 11 in CSF and n = 1 in serum, respectively). Histograms of each analyte were plotted and if the majority of intensities were ≤ 1, the analyte was excluded. This pertained to the analyte CFI HPA024061 (**Figure S4C, inset**) in both CSF and serum, which was hence excluded. Following these assessments, quality control was considered finished. Onwards only patients or controls with matched CSF and serum samples were investigated.

**Data Examination Following Quality Control**

Matched TBI patient and control samples were graphically examined per analyte and sample with regard to MFI_adjusted_, in order to examine validity of upstream quality control and compare our obtained analytes with both negative and positive controls. In order to ensure the validity of bi-compartmental assessments for both TBI and control patients, we examined patient sample and analyte correlations between serum and CSF (non-transformed data were used for calculations), using Spearman correlation. In addition, sibling antibodies were examined using Spearman correlation (non-transformed data) within compartments as well as the aforementioned between-compartment analyses. For sibling antibodies with within-compartment correlation Spearman ρ ≤ 0.2, the antibody sibling with the lowest between-compartment (i.e. CSF-serum) correlation was excluded from further bi-compartmental analyses. However, for mono-compartmental analyses, the sibling was included if the within-compartment correlation was deemed sufficient.

**Supplementary statistical analysis**

*Protein Characterization*

For tissue enrichment analysis, we employed the analytic criteria in the HPA (2) on the RNA Consensus data set, from which normalized expression (NX) values were retrieved. In brief, NX <1 was deemed as “not detected”. NX > 4 times other tissues’ minimal NX values (where NX_min_ ≥ 1) for a specific protein, *i*, was deemed as significantly enriched. If a protein was enriched in one region of an organ system but not another, we classified this as enriched within the organ system. The tissue with NX_max_ for a specific protein was deemed as the “highest enrichment tissue”. Within the Brain atlas, we calculated NX_mean_ for all proteins. CNS enrichment was defined in accordance with Equation 2 (Eq.2), where *i* denotes a specific protein. “Highest CNS tissue enrichment” was defined as the CNS tissue where a specific CNS enriched protein exhibited NX_max_. For visualizations, we used the upSetR package (3).

$${CNS enriched}_{i}={NX}_{i}> 4{NX}_{i, min} \cup{NX}_{i}> {NX}_{mean}$$

(Eq.2)

*Parallel assessments in CSF and serum*

We computed a correlation matrix of all included proteins and antibodies, as has been suggested (4). For each analyte we calculated the absolute median correlation with all other proteins and antibodies, compartment-wise as well as for all samples. If the absolute median correlation within both CSF, serum and the whole correlation matrix was < 0.3, that analyte was excluded. This pertained to n = 10 analytes (APP HPA001462, TMEM132D HPA010739, SLITRK1 HPA012414, PDYN HPA053342, GPR37L1 HPA064454, CSPG5 HPA071779, VSTM2B HPA073612, NPTX1 HPA077062, NTSR2 HPA077042, and TNF HPA077901). We examined suitability for dimensionality reduction analysis using Bartlett’s test of sphericity (5) (χ^2^ 82114 df(19900), p < 0.001) and the Kaiser Meyer Olkin (KMO) Measure of Sampling Adequacy (MSA) (5). The global MSA value was 0.62 (acceptable), and there were no individual KMO values < 0.075, which we used as cut-off.

*Proteomic/genotypic alterations and relationship with BBB disruption*

Cluster tendency was evaluated using the factoextra (6) and clustertend (7) package, both of which generated a Hopkins statistic H > 0.5. We chose clustering algorithm and number of clusters based on comparison between hierarchical, kmeans, and partitioning around mediods strategies, using the clValid (8) package. For CSF, we chose a hierarchical clustering strategy with 3 clusters, as this maximized the Dunn index. Employing a similar strategy for serum, the Dunn index was maximized for a hierarchical clustering regime with n = 2 clusters. For plotting, we presented the clustering algorithms using euclidean distance for dissimilarity and “ward.D2” as agglomeration method.

Supplementary Results

**Analyte profiles**

Analyte profiles (unique for one antibody-protein combination) were examined graphically. An example (protein C9, antibody HPA070709) is shown in **Figure S5A**. For comparison, we show a positive control (hIgG, **Figure S5B**) and a negative control (empty, **Figure S5C**). As can be seen, MFI values differed between compartments and patient groups for the specific analyte, whereas MFI values were homogenously high for hIgG and correspondingly low for empty (albeit some outlier values). Of note, different analytes exhibited very different MFI levels in general, as is shown for one TBI patient with matched samples in CSF (**Figure S5D**) and serum (**Figure S5E**).

Spearman correlations were assessed within patients and within analytes. Within patients, correlations between total protein MFI were in median (IQR) ρ 0.64 (0.59-0.67). Two examples (one control and one TBI patient) is shown in **Figure S6A-B**. In contrast, correlations between CSF and serum per analyte differed more. In **Figure S6C-D**, the protein FAM181B and MBP are shown for representative purposes. Whereas FAM181B shows acceptable correlation for its antibody HPA075523, its other antibody (HPA066861) exhibits a very low ρ = 0.066. In contrast, MBP shows different albeit high correlations for both its antibodies. For all analytes, median correlation (IQR) was ρ = 0.145 (0.06-0.23). A table depicting all analytes (protein/antibody combinations) where ρ < 0 is included (**Table S2**). For proteins represented by one mere antibody, it is impossible to know the source of these low correlations. It could be due to blood-brain barrier integrity and low BBB-dependent passage but it might also depend on protein features, such as low antigen detection in serum due to the more complex serum composition. In contrast, for proteins represented by multiple antibodies, i.e. *sibling antibodies*, one might assume that if one antibody is well correlated between CSF and serum for a protein, whereas the other antibody is not, then that particular antibody is suboptimal and should be excluded. With regard to this, we examined the 42 sibling antibodies in our material.

Two pairs of sibling antibodies and their within-compartment correlations are depicted in **Figure S6E-H**. As can be seen, whereas the protein C9 correlates very well in both serum and CSF between siblings, the brain-enriched protein AQP4 has a low correlation in serum ρ = 0.059 in spite of having an excellent correlation in CSF (ρ = 0.996). This highlights that some antibodies are probably ineligible to analyze in serum, rather than that the antibody per se is flawed. In order not to exclude proteins that might be of key interest in the CSF compartment (such as AQP4), we calculated the within-compartment sibling Spearman correlations. For proteins with ρ ≤ 0.2, we excluded the antibody that showed the lowest between-compartment correlation per sibling antibody pair. This means that in **Figure S6C**, we exclude FAM181B HPA066861, while retaining FAM181B HPA075523 for subsequent analyses. This resulted in exclusion of n = 9 sibling antibodies (OLIG1 HPA077730, IL4 HPA042270, MEPE HPA038004, AQP4 HPA014784, MBP HPA073581, FAM181B HPA066861, CASKIN1 HPA055990, NCAN HPA036814, GPR37L1 HPA052631).

**Protein Characterization**

Of all proteins included, n = 45 proteins (25%) were not characterized on the protein level but merely on the RNA level in the HPA. In the Brain Atlas, n = 3 (1.7%) of our proteins (VEGFC, NEFL, CCL18) were not characterized. In total, n = 17 (9.6%) proteins had no described function within the HPA. Of note, all of the data incorporated from the Human Protein Atlas comprise healthy tissue protein expression, why this not per se pertains to injury state.

Supplementary Figure Legends

**Supplementary Figure 1: Sample acquisition time and preanalytical handling time differed between patients.**

CSF samples were obtained at 60.8 hours (36.6-109.1) following trauma, whereas serum samples were obtained 53.3 hours (30.5-91.1) following trauma (**A**). The discrepancy is due to the fact that among the originally included TBI patients, n = 96 had solely serum sampled. Following acquisition, samples were stored in a local biobank at 4° C until delivery to the study center biobank. Local biobank/fridge storage was in median 1 day (0-1) for both CSF and serum samples (**B**). **Abbreviations:** CSF, cerebrospinal fluid.

**Supplementary Figure 2: Total protein content was similar across different storage times.**

Total protein content categorized depending on local storage time at 4° C was assessed per patient sample (**A**) and per analyte, of which one representative example is shown (**B**). Graphically, there were no discernible differences between storage time and total protein content. **Abbreviations:** CSF, cerebrospinal fluid; MFI, median fluorescence intensity.

**Supplementary Figure 3: Bead count quality control excluded a few patients but no analytes.**

Using a suspension bead antibody array, quality control operations initially comprised bead count assessment across all patient samples (**A**) and analytes (**B**). In total, n = 4 TBI samples had bead count < 30 and hence, n = 4 patients (n = 8 samples) were excluded from subsequent analyses. All analytes exhibited median bead count ≥ 30, why no analyte exclusion was conducted. Abbreviations: CSF, cerebrospinal fluid; TBI, traumatic brain injury. Full protein names are given in **Table S1**.

**Supplementary Figure 4: MFI assessment resulted in background subtraction as CSF samples generally exhibited higher MFI values as well as exclusion of one analyte.**

MFI was examined per sample (**A**), whereupon it became evident that CSF samples (notably blank CSF samples and pooled CSF samples) exhibited higher MFI values than their serum equivalents. To overcome this systematic bias, background was executed with improved results (**B**). In **A** and **B** the inset depicts a magnification of the blank and pooled samples groups. Following background subtraction, MFI per analyte was examined (**C**). A few analytes graphically exhibited MFI values very close to those of negative controls (see for example APP and rIgG). Analytes (n = 11) exhibiting MFI values lower than median MFI values for rIgG were examined further using histograms. If the majority of intensities were ≤ 1, the analyte was excluded, resulting in the exclusion of CFI HPA024061 (**C, inset**). **Abbreviations:** Blank C, blank CSF sample; Blank S, blank serum sample; CSF, cerebrospinal fluid; MFI, median fluorescence intensity; Pool C, pooled CSF samples; Pool S, pooled serum samples. Full protein names are given in **Table S1**.

**Supplementary Figure 5: Assessment following quality control operations showed methodological reliability across patients and analytes.**

Analyte profiles were examined following quality control operations on matched samples (n = 1 CSF sample and n = 1 serum sample per n = 90 TBI patients and n = 15 control subjects). Individual analytes exhibited clear MFI value differences across matrix (CSF/serum) and sample (TBI/control), of which one representative example (C9 HPA070709) is shown (**A**). Notably, the MFI values were clearly discrepant from positive control values (**B**) and negative control values (**C**). Please note the different y axis scales on panels **A-C**. Across study subjects, MFI values of individual analytes were different both depending on analyte but also depending on matrix (CSF/serum), of which one TBI patient is shown (**D-E**). **Abbreviations:** CSF, cerebrospinal fluid; MFI, median fluorescence intensity. Full protein names are given in **Table S1**.

**Supplementary Figure 6: Patient samples correlated between CSF and serum, whereas individual analytes exhibited a variable CSF/serum correlation.**

Among individually matched patient samples the median correlation was ρ = 0.64 (0.59-0.67), of which one control subject (**A**) and one TBI patient (**B**) are shown. When analyzing all individual analytes per compartment, median correlation was ρ = 0.145 (0.06-0.23). This finding was due to some analytes having very low CSF-serum correlations (**C**), whereas other analytes exhibited stable CSF-serum correlations (**D**). For proteins represented by one antibody, this was difficult to assess further. In contrast, for sibling antibodies, sibling correlations within compartments were assessed (**E-H**). As demonstrated in **G-H**, AQP4 has excellent sibling correlation in CSF but low in serum. One tentative explanation for this is that some antibodies had a low detection rate in serum. This resulted in additional analyte exclusion. **Abbreviations:** CSF, cerebrospinal fluid; MFI, median fluorescence intensity; TBI, traumatic brain injury. Full protein names are given in **Table S1**.

**Supplementary Figure 7: Missing values.**

Missing values are depicted. Of note, not all variables could be collected for control subjects, which does not represent true missing values. An example of this is GOS, which for obvious reasons cannot be collected for a healthy control. Many missing values are seen among the variables s-albumin #, csf-albumin #, and their corresponding time values. This depends on the fact that clinical sampling of albumin occasionally occurred at multiple time points and the albumin sampling time point in closest time to proteomic sampling time point was used, thus yielding more missing values than used for down-stream analysis. As none of the crucial variables had missing data exceeding 30 % of all TBI patient observations, no imputation/compensation for missing values was attempted. **Abbreviations:** AIS, Abbreviated Injury Severity Score; CSF, cerebrospinal fluid; CT, computerized tomography; GCS, Glasgow Coma Scale; GOS, Glasgow Outcome Score; ISS, injury severity score; S-, serum-; Q_A_, albumin quotient; TBI, traumatic brain injury.

**Supplementary Figure 8: Relative Protein Abundance under Healthy Conditions.**

Among healthy controls, the relative abundance of proteins in CSF and serum were compared using significance threshold |log_2_ FC| > 1 and adjusted p-value < 0.01. Non-significant values are diminished in size and shaded light-gray. As can be seen, nervous system proteins have a higher abundance in CSF compared with serum (log_2_ FC > 1), whereas neuroinflammatory proteins have a higher abundance in serum (log_2_ FC < 1). **Abbreviations:** CSF, cerebrospinal fluid; FC, fold change. Full protein names are given in **Table S1**.

**Supplementary Figure 9: Protein Pathway Alterations in the Validation Cohort.**

Pathway upregulation in the validation cohort in serum following TBI. Similarly to the matched TBI cohort, complement pathways were upregulated. Full protein names are given in **Table S1**.

Supplementary References

1. Jobling JW, Eggstein AA, Petersen W. Serum proteases and the mechanism of the abderhalden reaction. Studies on ferment action. xx. J Exp Med. 1915 Mar 1;21(3):239–239.

2. Collaborators. HPA. Assays and annotation - The Human Protein Atlas [Internet]. [cited 2020 Apr 29]. Available from: https://www.proteinatlas.org/about/assays+annotation

3. Gehlenborg N. UpSetR: A More Scalable Alternative to Venn and Euler Diagrams for Visualizing Intersecting Sets. Comprehensive R Archive Network (CRAN); 2019.

4. Field A, Miles J, Field Z. Discovering statistics using R. London: SAGE Publications Ltd; 2012.

5. Revelle W. Procedures for Psychological, Psychometric, and Personality Research [R package psych version 1.9.12.31]. Comprehensive R Archive Network (CRAN); 2018.

6. Kassambara A, Mundt F. factoextra: Extract and Visualize the Results of Multivariate Data Analyses. Comprehensive R Archive Network (CRAN); 2019.

7. Luo Y, Zeng R. clustertend: Check the Clustering Tendency. R package; 2015.

8. Brock G, Pihur V, Datta S, Datta S. clValid : An R Package for Cluster Validation. J Stat Softw. 2008;25(4).
